# Phylogeography and population genetics of the endemic Malagasy bat, *Macronycteris commersoni* s.s. (Chiroptera: Hipposideridae)

**DOI:** 10.7717/peerj.5866

**Published:** 2019-01-17

**Authors:** Andrinajoro R. Rakotoarivelo, Steven M. Goodman, M. Corrie Schoeman, Sandi Willows-Munro

**Affiliations:** 1 Department of Zoology, University of Venda, Thohoyandou, Limpopo, South Africa; 2 School of Life Sciences, University of Kwa-Zulu Natal, Pietermaritzburg, Kwa-Zulu Natal, South Africa; 3 Natiora Ahy, Antananarivo, Madagascar; 4 Field Museum of Natural History, Chicago, IL, United States of America; 5 Association Vahatra, Antananarivo, Madagascar; 6 School of Life Sciences, University of Kwa-Zulu Natal, Westville, Kwa-Zulu Natal, South Africa

**Keywords:** Diversification, Geographical structure, Bioclimate, Madagascar, *Macronycteris commersoni*

## Abstract

*Macronycteris commersoni* (Hipposideridae), a bat species endemic to Madagascar, is widespread across the island and utilizes a range of habitat types including open woodland, degraded habitats, and forested areas from sea level to 1,325 m. Despite being widely distributed, there is evidence that *M. commersoni* exhibits morphological and bioacoustic variation across its geographical range. We investigated the fine-scale phylogeographic structure of populations in the western half of the island using extensive spatial sampling and sequence data from two mitochondrial DNA regions. Our results indicated several lineages within *M. commersoni.* Individuals collected from northern Madagascar formed a single monophyletic clade (clade C). A second clade (clade B) included individuals collected from the south-western portion of the island. This second clade displayed more phylogeographical partitioning with differences in mtDNA haplotypes frequency detected between populations collected in different bioclimatic regions. Lineage dispersal, genetic divergence, and timing of expansion events of *M*. *commersoni* were probably associated with Pleistocene climate fluctuations. Our data suggest that the northern and the central western regions of Madagascar may have acted as refugia for this species during periods of cooler and drier climate conditions associated with the Pleistocene.

## Introduction

Madagascar has been isolated from other continental landmasses for over 120 million years (Ali & Aitchison, 2008). As a consequence, the biota is unique with numerous endemic higher-level taxonomic groups, genera and species (Myers et al., 2000). The mechanisms driving this diversity are varied. Excluding vicariance, which can be applied to some extant vertebrate lineages (Noonan & Chippindale, 2006; Yoder & Nowak, 2006), different abiotic (e.g., ocean current direction or prevailing winds) and biotic (e.g., dispersal ability) filters have limited or promoted the diversification of taxa across the island (Ali & Huber, 2010; Samonds et al., 2012; Samonds et al., 2013). Examples of patterns of extensive speciation and morphological variation in volant vertebrates include birds of the families Vangidae and Bernieridae (Cibois et al., 2001; Jønsson et al., 2012; Reddy et al., 2012). In contrast, other adaptive radiations on the island show distinctly less morphological differentiation and these groups contain numerous cryptic species, for example, bats of the genus *Miniopterus* (Christidis et al., 2014; Schoeman et al., 2015). Further, there are cases of presumed congenerics colonizing the island independently of one another, for example bats of the family Molossidae (Lamb et al., 2011) and Rhinonycteridae (Foley et al., 2015; Russell et al., 2007). Regardless of the mode of speciation, the periods of rapid cladogenesis among these different groups are not coincidental, and no single unifying explanation has been presented to explain successful colonization and subsequent diversification patterns amongst extant volant Malagasy vertebrates (Samonds et al., 2012; Samonds et al., 2013).

**Figure 1:**
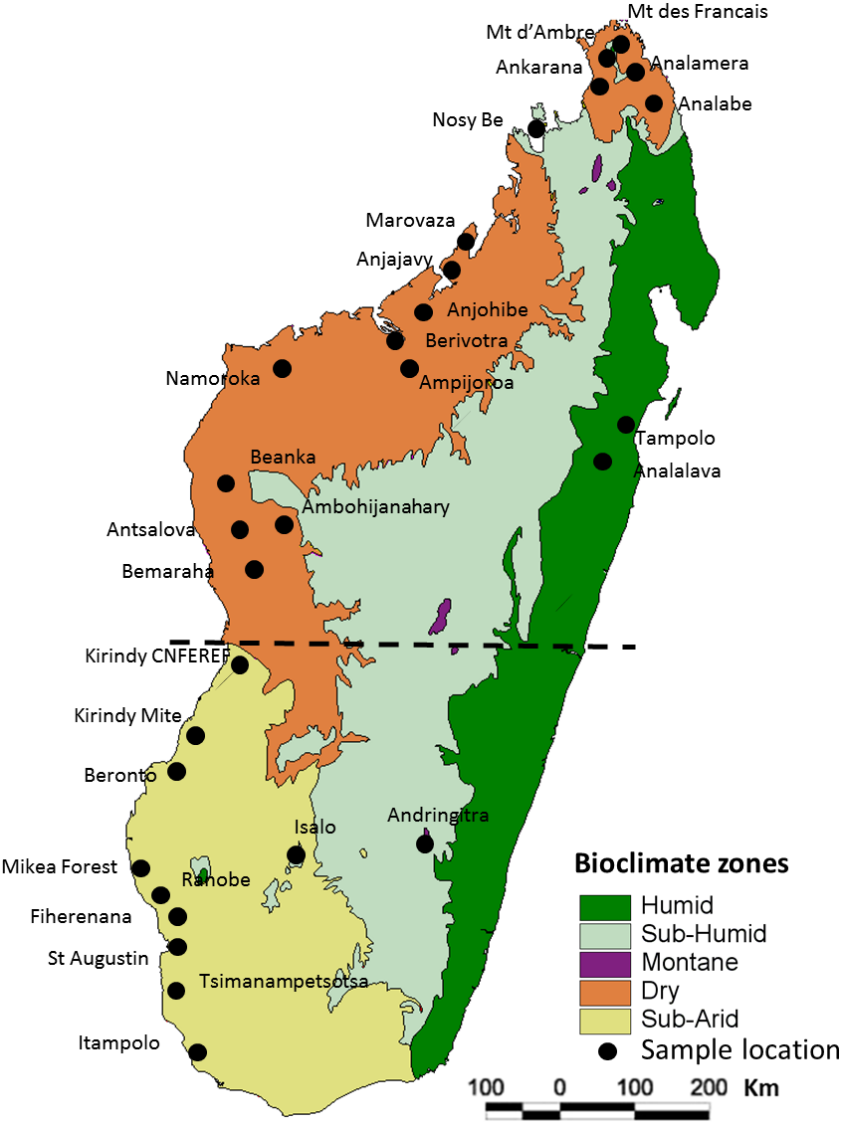
Map of Madagascar showing the different collection localities of specimens of *Macronycteris commersoni* used in this study. The map overlay is the simplified bioclimatic zones classification proposed by Cornet (1974). The stippled line separates the “northern group” from the “southern group”.

Factors that may mediate colonization success include the period of initial colonization, ranging from the Mesozoic through the Cenozoic (Holocene), and life-history traits (e.g., large organisms with fast and efficient flight are more likely to colonize than small and slow flying organisms). Subsequent biogeographic and phylogeographic patterns of lineage turnover are driven in part by the landscape and climatic heterogeneity of the island (Pearson & Raxworthy, 2009; Vences et al., 2009; Wilmé, Goodman & Ganzhorn, 2006). Compared to large tropical islands such as Borneo, the major biomes of Madagascar exhibit sharp geographical limits (Vences et al., 2009). The western coastal region is particularly variable and includes three different bioclimatic zones (Fig. 1) and this aspect can explain the biogeographic patterns of some mammalian taxa (Martin, 1972).

Differences in modes of dispersal and habitat requirements amongst flying Malagasy vertebrates have resulted in different biogeographic and phylogeographic patterns (e.g., for birds see Cruaud et al., 2011; Fuchs et al., 2007; Fuchs et al., 2013; for bats see (Chan et al., 2011; Goodman et al., 2010a; Goodman et al., 2010b; Goodman et al., 2016; Lamb et al., 2012; Ratrimomanarivo et al., 2007; Ratrimomanarivo et al., 2008; Ratrimomanarivo et al., 2009a; Ratrimomanarivo et al., 2009b; Russell et al., 2007; Russell, Goodman & Cox, 2008; Russell et al., 2008; Weyeneth, Goodman & Ruedi, 2011). Madagascar is an excellent model system for testing and contrasting the process of species diversification and fine-scale spatial patterning across different lineages.

*Macronycteris commersoni* (Family Hipposideridae), feeds predominantly on Coleoptera, and is widespread across Madagascar, utilizing a wide variety of habitat types including open woodland, degraded habitats, and forested areas from sea level to 1,325 m (Goodman & Ramasindrazana, 2013; Rakotoarivelo et al., 2007; Rakotoarivelo et al., 2009; Razakarivony, Rajemison & Goodman, 2005). It occupies day roosts in caves found in areas of eroded sedimentary rock, often forming colonies of several thousand individuals. Individuals also roost under vegetation in areas of pristine and degraded forest vegetation (Goodman, 2006; Raharinantenaina et al., 2008). There is evidence that *M. commersoni* shows morphological and bioacoustic variation across its geographical range. Both of these parameters show a clinal pattern correlated with latitude (Ranivo & Goodman, 2007; Ramasindrazana et al., 2015). However, seasonal intra-island movements of *M*. *commersoni* has been documented in south-western Madagascar (Rakotondramanana & Goodman, 2011; Ramasindrazana et al., 2015). For details on the complex taxonomic history of *M. commersoni* sensu lato, which previously included some African populations, see Goodman et al. (2016). Herein, we consider this species endemic to Madagascar.

Recent molecular work including specimens referred to as *M*. *commersoni* from different areas of Madagascar, particularly the western half of the island, found several independently evolving lineages, some geographically structured (Rakotoarivelo et al., 2015). On the basis of these results, a cryptic endemic species (clade A in (Rakotoarivelo et al., 2015) was identified, and subsequently named as *M*. *cryptovalorona* (Goodman et al., 2016). Rakotoarivelo et al. (2015) found *M*. *commersoni* sensu stricto (clades B and C therein) to be sister to African *M*. *vittatus* and *M*. *gigas*, with *M*. *cryptovalorona* basal to this grouping. On the basis of molecular clock estimates, clade A diverged from clades B and C during the Miocene, approximately 5.81 MYA and clades B and C last shared a common ancestor about 3.38 MYA. Two hypotheses were proposed to explain colonization of Madagascar by large-bodied *Macronycteris.* First, clade A and clade B-C could have originated from two independent eastward dispersal events from Africa. The second hypothesis involves multiple, bidirectional dispersal, with an early eastward dispersal to Madagascar, followed by a later back-dispersal to Africa.

Herein we focus on intra-population variability within *M. commersoni* sensu stricto, specifically clades B and C of Rakotoarivelo et al. (2015) across Madagascar. Using sequence data from two mitochondrial genes and extensive geographical sampling, we investigate the fine-scale phylogeographic history and relationships among populations of *M. commersoni* occurring in western lowland Madagascar to determine if the distinct regional bioclimatic zones, which are known to have promoted diversification in other taxa (Martin, 1972), have also shaped population structure of this species.

## Materials & Methods

### Sample collection

All of the samples used herein were associated with specimens deposited in museums (Table S1) and no individual was specifically collected for this study. In total, 146 specimens of *Macronycteris commersoni* falling within clades B and C of Rakotoarivelo et al. (2015) and Goodman et al. (2016), from 29 localities were included. These samples span the western latitudinal length of the island, including all the major bioclimatic zones. The sample set includes 140 specimens from the dry and subarid bioclimatic zones, five specimens from the humid or subhumid bioclimatic zones (Montagne d’Ambre, Nosy Be, Tampolo, Analalava, Andringitra), and one specimen from the mid-western Central Highlands at the limit of the subhumid zone (Ambohijanahary) (Fig. 1; Table S1). Two African species, *M*. *gigas* and *M*. *vittatus,* were included as out-group taxa and used to root the phylogenetic trees.

### DNA extraction and amplification

Genomic DNA was isolated using the NucleoSpin^®^ Tissue kit (Macherey-Nagel, Düren, Germany), following the manufacturer’s protocol for tissue samples. Two mitochondrial (mtDNA) markers were amplified: hypervariable control region (CR, 481 bp) using the primers P/E (Wilkinson & Chapman, 1991) and cytochrome b (*Cyt b*, 705 bp) using the primers JorF/H15553 (Irwin, Kocher & Wilson, 1991; Rakotoarivelo et al., 2015). PCR amplifications consisted of: ∼20–150 ng template DNA, 2.5 µl 10×  KAPA buffer, 1 U KAPA Taq DNA polymerase (Kapa Biosystems, Wilmington, MA, USA), 200 µM dNTPs, 0.2 µM of each primer and 18.4 µl dH_2_O to give a final reaction volume of 25 µl. The PCR cycle parameters included an initial denaturation step at 95 °C for 3 min followed by 30 cycles at 95 °C for 30 s, 50–55 °C for 30 s, 72 °C for 30 s, with a final extension step at 72 °C for 5 min. PCR reactions included a negative control to check for possible contamination. PCR products were sent to the Central Analytical Facility at Stellenbosch University, South Africa, for sequencing. Cycle sequencing was performed using the BigDye Chemistry, v3.1 and sequencing products were analyzed on an Applied Biosystems 3730xl Genetic Analyzer (Applied Biosystematics, Perkin Elmer, Waltham, MA, USA). All sequences were first aligned using ClustalW (Thompson et al., 1997) as implemented in BioEdit (Hall, 1999), and thereafter manually optimized. All new sequences were deposited in GenBank (Table S1).

### Phylogenetic analyses and molecular clock dating

The two markers (CR, *Cyt b*) were analyzed separately and then combined into a single data set. The number of variable sites, number of parsimony informative sites and nucleotide frequencies were estimated for each data set in MEGA 6 (Tamura et al., 2013).

**Figure 2:**
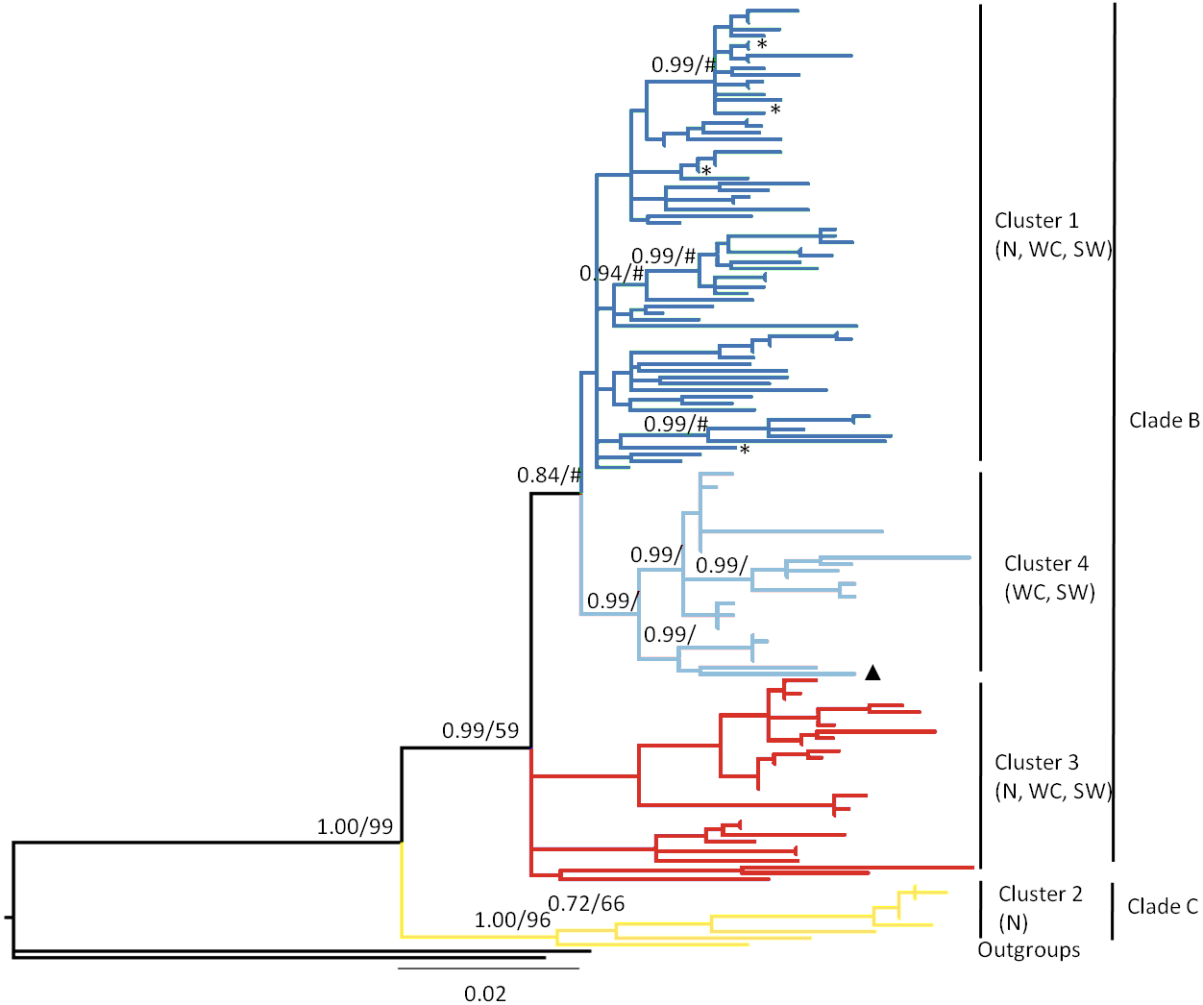
Bayesian phylogram based on the combined analysis of mtDNA control region and cytochrome *b* data drawn from 146 *Macronycteris commersoni* individuals. Nodal support values are represented as Bayesian posterior probability/maximum likelihood bootstrap support. Only values greater than 50 are shown. The tree is color coded based on the result of the genetic mixture analysis in BAPS (Fig. 4). ∗, specimens from eastern Madagascar, specifically the sites of Analalava and Tampolo; ▴, specimen from Andringitra; N, north; WC, west central; SW = southwest.

Phylogenetic reconstruction was performed using both maximum likelihood (ML) and Bayesian (Bayes) approaches in the programs Garli 2.0 (Zwickl, 2006) and MrBayes 3.2 (Ronquist et al., 2012), respectively. The most appropriate substitution model for each gene (CR—GTR+I+G, *Cyt b*—TrN+I+G; Fig. 2) was selected using the Akaike information criterion (AIC) as implemented in jModelTest (Darriba et al., 2012; Posada & Crandall, 1998). For the concatenated data set, partitioned analyses were conducted, with data partitioned by gene. The parameters of nucleotide substitution models were unlinked across partitions. Each ML analysis was initiated from a random starting tree, with nodal support assessed using 1,000 bootstrap replicates. Two independent Bayes runs of 5 million generations each were performed; each run consisted of four Monte Carlo Markov chains (MCMC), with topologies sampled every 500 generations. The program Tracer 1.6 (Rambaut et al., 2014) was used to determine that the effective sample size (ESS) had reached >200 for all parameters. A 50% majority rule consensus tree was constructed using the CONSENSE program in the PHYLIP package (Felsenstein, 2005). In each simulation, the first 20% of generations were discarded as burn-in.

We assessed levels of allele sharing among sampling localities and biomes, using haplotype networks. Since loci located in the mitochondrial genome are tightly linked, we concatenated mtDNA sequences and reconstructed a single mitochondrial gene network. We used the MP method in Haploviewer (http://www.cibiv.at/g˜reg/haploviewer; Salzburger, Ewing & Von Haeseler, 2011) to calculate and draw the resulting networks.

The *Cyt b* data was used to estimate the time of most recent common ancestor of major evolution lineages because of its moderate mutation rate. TMRCA was assessed using BEAST (Drummond & Rambaut, 2007) with a strict molecular clock, a coalescent prior (appropriate for intraspecific radiations), and the GTR + I + G model. A fixed mean substitution rate of 1. 30 × 10 −8 subs/site/year (Nabholz, Glemin & Galtier, 2008; Thong et al., 2012; Puechmaille et al., 2012; Rakotoarivelo et al., 2015; Liu et al., 2016) was applied as a fixed mean substitution rate. Several preliminary short runs were performed to optimize the prior parameters, including models and MCMC length, and to ensure sufficient mixing of chains. Tracer 1.6 was used to assess the convergence of MCMC chains (Rambaut et al., 2014). We ran three independent runs of 20 million generations, with sampling every 1,000 generations, with a burn-in of the first 20% of generations. Results were combined using Tracer 1.6 (Rambaut et al., 2014); effective sample size (ESS) values exceed 200 for all parameters.

### Population structure analyses

To examine the fine-scale population structure of *M*. *commersoni*, without making *a priori* assumptions about the partitioning of genetic diversity, a Bayesian model-based approach inferring hidden genetic population structures was implemented in the program BAPS 6 (Bayesian analysis of population structure; Cheng et al., 2013; Corander & Marttinen, 2006. Analyses were first performed on the entire dataset (including all sequenced individuals from the latitudinal range of *M*. *commersoni*) and then repeated on subsections of the data, including only individuals assigned to the “northern group” and the “southern group” (see results; Fig. 1). In each independent run the number of proposed clusters (K) ranged from 1 to 10, with five runs for each K. In each case, analyses were conducted using the concatenated mtDNA.

A Mantel test was used to determine the relationship between genetic and geographic distance of *M. commersoni* samples. Significance was assessed by 1,000 permutations using the Alleles In Space (AIS) program (Miller, 2005).

Genetic differentiation was evaluated using analysis of molecular variance (AMOVA) in Arlequin 3.5 (Excoffier & LischerH, 2010). Population structure was assessed at three hierarchical levels of subdivision (among regions, among populations within regions, and within populations). The northern and southern groups were separated by the boundary between the subarid and dry bioclimatic zones (Fig. 1). To evaluate possible correlations between genetic differentiation and bioclimatic aspects of Madagascar, we also used AMOVA to test significant genetic differentiation among four bioclimatic zones, following the classification of Cornet (1974): “Dry1” includes sites from Nosy Be to the northern most locality; “Dry2” from Marovaza to Bemaraha; “Subarid”; and “Humid-Subhumid” as delineated in Fig. 1.

### Demographic analysis

Demographic analyses were performed separately for *M. commersoni* clades (clades B and C, see results) using the *Cyt b* data. In addition to Tajima’s D (Tajima, 1989) and Fu’s Fs (Fu, 1997), that may be used to infer demography in neutrally evolving loci, demographic changes in both clades were also inferred from the observed mismatch distribution for each of the clades, using the raggedness index (R2; Harpending, 1994; Ramos-Onsins & Rozas, 2002) according to the population expansion model in DnaSP version 5.10 (Librado & Rozas, 2009). This measure quantifies the smoothness of the observed mismatch distribution, with lower raggedness values indicating populations that have experienced sudden expansion, whereas higher raggedness values suggest stationary or bottlenecked populations (Harpending et al., 1993; Harpending, 1994). Lastly, changes in effective population size were inferred using Bayesian Skyline Plots (BSP; Drummond et al., 2005). These plots utilize the coalescent properties of gene trees to plot population size changes over time, and were inferred using BEAST (Drummond & Rambaut, 2007). A mitochondrial substitution rate of 1. 30 × 10 −8 subs/site/year was used. The lengths of the MCMC chains were set to 20 million to achieve effective sample sizes (ESS) and proper mixing of Markov chains. To account for biases due to genetic structure (Ho & Shapiro, 2011), we divided the data into clades B and C and reconstructed separately their demographic history.

## Results

### Genetic diversity and divergence

The nucleotide composition and levels of variation of the two mitochondrial genes differed; CR had the highest number of variable characters (132 variable sites), while *Cyt b* was more conserved (76 variable sites). The CR partition contained the highest number of parsimony informative characters (91 parsimony informative sites), whereas *Cyt b*, contained 52 parsimony informative characters (Table 1).

**Table 1:** Characteristics of mtDNA datasets used in this study of Macronycteris commersoni . Patterns of sequence variability are presented for two mtDNA regions (CR and *Cyt b*) and the combined data matrix. The total number of nucleotide sites, variable and parsimony informative sites, as well as nucleotide frequencies are given for each partition and the combined data matrix.

Gene	Total number of individuals	Total sites	Variable sites	Parsimony informative sites	Nucleotide frequencies			
					%A	%T	%C	%G
CR	146	481	132	91	32.90	27.00	25.80	14.34
*Cyt b*	146	703	76	52	26.90	27.36	30.57	15.18
Combined	146	1,184	208	143	29.90	27.18	28.18	14.76

For the CR, DnaSP analyses identified 92 unique haplotypes. The haplotypic diversity for this dataset was high (Hd = 0.98), but the nucleotide diversity was low (*p* = 0.032). For the *Cyt b* gene, the same analysis identified 70 unique haplotypes, also with high haplotypic diversity (Hd = 0.98) and low nucleotide variability (*π* = 0.008).

### Phylogeny and time of most recent common ancestor

Maximum likelihood and Bayesian analyses produced consistent topologies. There was no significant conflict between the CR and *Cyt b* topologies, although most clades in phylogenetic trees generated from the CR data had low posterior probability (<0.60) and bootstrap support (<50%) values (Fig. S1). Consequently, the Bayesian phylogram constructed from the concatenated mtDNA data set is presented as Fig. 2.

Both ML and Bayesian analysis of the concatenated data matrix (CR + *Cyt b*; Fig. 2) recovered all Malagasy *M. commersoni* as a single monophyletic lineage, supporting the conclusions of Rakotoarivelo et al. (2015). Within the species, the two clades suggested by Rakotoarivelo et al. (2015) were again recovered: clade B (ML bootstrap, 59; Bayes’ PP, 0.99) and clade C (ML bootstrap, 96; Bayes’ PP, 1.0), although the level of phylogenetic resolution was much higher in clade C and shallower in clade B. Samples belonging to clade C were all collected in the northern portion of the island. Clade B is much more geographically wide spread and included specimens collected from the north (N), west-central (WC) and south-west (SW) parts of the island. The phylogenetic analysis did not support differentiation based on bioclimatic zones.

**Figure 3:**
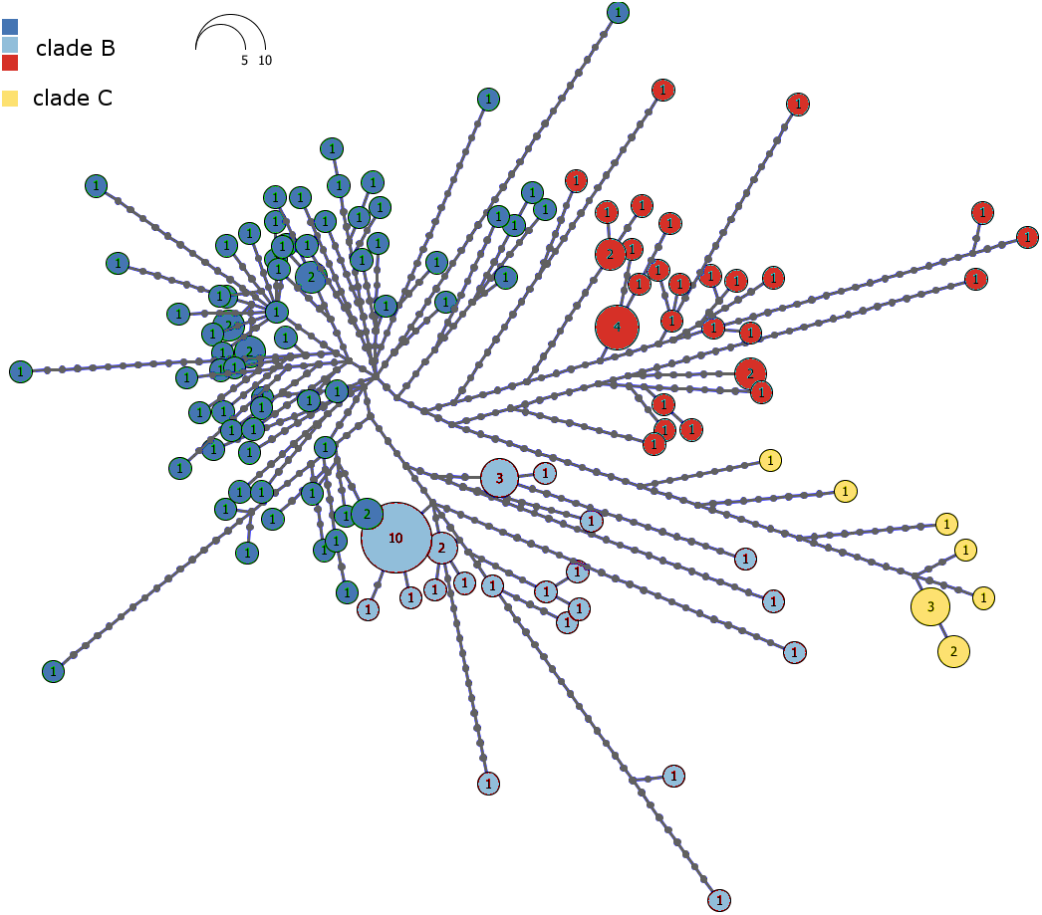
Haplotype network of the combined mtDNA control region and cytochrome b data drawn from 146 individual *Macronycteris commersoni*. Haploviewer was used to estimate the most parsimonious network. Clades are color coded based on the BAPS clustering results (as in Fig. 2). Numbers inside the proportionally sized circles represent the number of individuals sharing that particular haplotype.

The haplotype network of Malagasy *M*. *commersoni* confirms the split between clade B and C (Fig. 3). Clades B and C were separated by 13 mutational steps, whereas haplotype differences within clades were smaller (Fig. 3).

**Figure 4:**
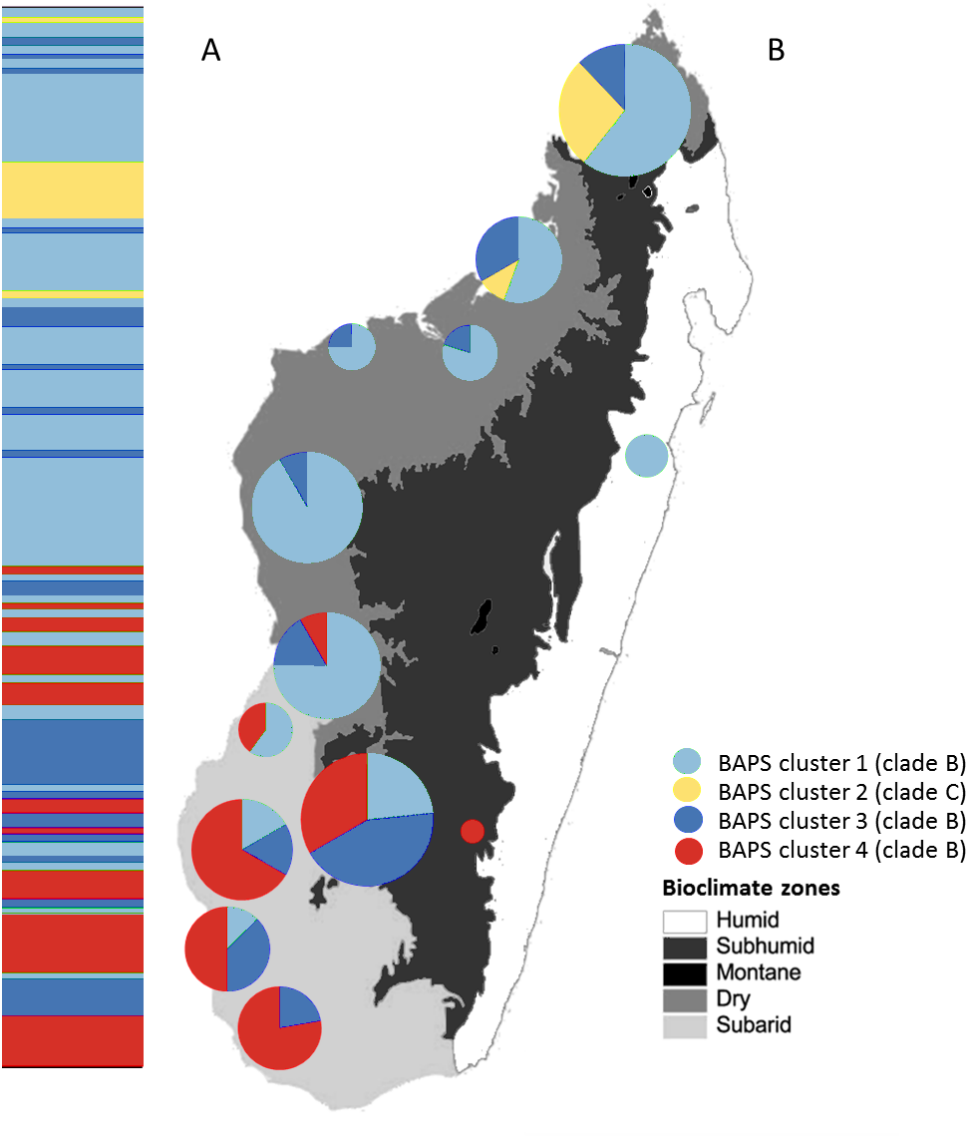
Posterior mode clustering of *Macronycteris commersoni* using the concatenated mtDNA data in an individual-based genetic mixture analysis in BAPS. (A) Bar graphs for genetic structure with *K* = 4. Each bar represents an individual organized by latitude. Every different colour represent a cluster. (B) Distribution of estimated BAPS cluster frequencies. The 146 specimens are grouped by latitude and by locality and some neighbouring localities have been grouped together.

Molecular clock analysis suggests that the most recent common ancestor of *M. commersoni* could be traced back to 0.82 (95% highest posterior density (HPD); 0.48–1.12) MYA. The TMRCA estimates suggest that the two sister clades B and C shared a common ancestor in the mid-Pleistocene. The TMRCA estimates obtained for individuals belonging to clade B were 0.70 (95% HPD; 0.42–0.89) MYA. For clade C, the TMRCA was 0.32 Ma (95% HPD; 0.13–0.46) MYA. These dates indicate that the major divergence events occurred in the mid to late Pleistocene.

### Bayesian clustering and population structure

The Bayesian clustering method performed on the concatenated sequence data defined four genetically distinct clusters (*P* = 1, optimal partition, log likelihood = −5536.8; Fig. 4). These included two widespread clusters distributed throughout the range of *M*. *commersoni* (cluster 1 and cluster 3), one cluster that contained only individuals from the north (cluster 2), and a cluster restricted to the subarid bioclimatic zone (cluster 4). Cluster 2 in the BAPS analysis includes all individuals from clade C recovered by the phylogenetic analysis. The other clusters were also supported by the phylogenetic analysis, but with weak nodal support values.

**Figure 5:**
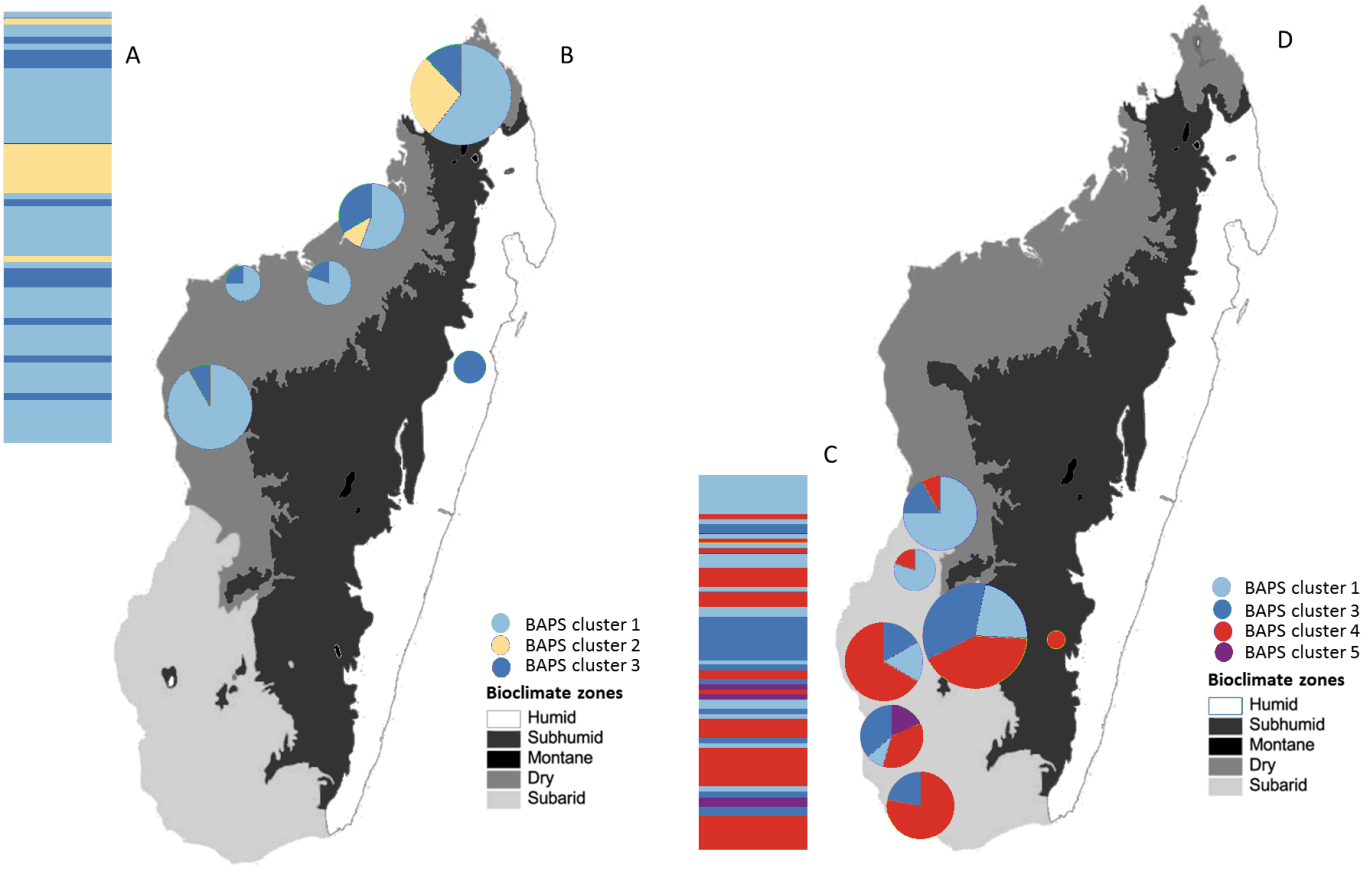
Posterior mode clustering of the northern group and the southern group of *Macronycteris commersoni* using individual-based genetic mixture analysis in BAPS. Bar graphs for genetic structure respectively for both group (A with K = 3 and C, K = 4). Individuals in the northern group (69) and southern group (77) are grouped by latitude and locality. Proportional membership of each *M. commersoni* in the genetic clusters inferred by BAPS for (B) the northern group and (D) the southern group. Distributions of the estimated BAPS cluster frequency for (B) the northern group and (D) the southern group are shown.

Additional phylogeographic resolution was recovered when southern and northern groups were analyzed independently (Fig. 5). Four genetically distinct BAPS clusters were recovered within the southern group (*P* = 0.99, log likelihood of optimal partition = −2295.82), whereas within the northern group, three distinct genetic clusters were recovered (*P* = 1, log likelihood of optimal partition = −2836.21). When all individuals were used, the Mantel test failed to support the isolation-by-distance (IBD) model (*r* =  − 0.009, *P* > 0.05).

Analysis of molecular variance (AMOVA) revealed that significant genetic structure was present at all three hierarchical levels (regions, populations within regions, and within populations). However, most of the total variation was found within populations (96.41% for geographical group and 94.62% for bioclimatic group, Table 2). The subarid bioclimatic region was the most genetically differentiated (Table 3). There was also significant differentiation between the Dry1 and Dry2 zones but there was no significant differentiation between the dry regions (Dry1 and Dry2) and the humid-subhumid region (Table 3).

**Table 2:** Analyses of molecular variance (AMOVA) for mtDNA of Macronycteris commersoni grouped by regions and by bioclimatic zones. Percentage of genetic variation and *P*-values significance attributable to heterogeneity among groups, among populations within regions, within populations are presented in each column. Statistically significant results were indicated by asterisks (^∗^*P* < 0.05; ^∗∗^*P* < 0.01; ^∗∗∗^*P* < 0.001).

Population groups	Among groups	Among populations within regions	Within populations
Northern/Southern	1.27^∗∗∗^	2.32^∗∗^	96.41^∗∗∗^
Dry1/Dry2/Subarid/ Humid-Subhumid	2.82^∗∗∗^	2.56^∗^	94.62^∗∗∗^

**Table 3:** Genetic differentiation among bioclimatic regions. Pairwise  values for combined mtDNA data among bioclimatic regions are presented to the left of the diagonal. Statistically significant results were indicated by asterisks (^∗^*P* < 0.001).

	Dry1	Dry2	Subarid	Humid-Subhumid
Dry1	–			
Dry2	0.064^∗^	–		
Subarid	0.151^∗^	0.069^∗^	–	
Humid-Subhumid	0.050	0.0617	0.155^∗^	–

### Historical demography

Mismatch distribution analysis based on *Cyt b* revealed that the various *M. commersoni* clades differ in demographic history. Clade B returned significant negative Fu’s Fs and Tajima’s D values, rejecting the neutrality/constant population size null hypothesis, indicating an expanding population. This finding is corroborated by the low R2 value and unimodal mismatch distribution that are also indicative of population expansion. In contrast, Fu’s Fs and Tajima’s D values for clade C failed to reject the neutrality/constant population size null hypothesis (Table 4). This coupled with the high R2 value and bimodal mismatch distribution suggests that clade C represents a stable population.

**Table 4:** Results of neutrality tests and mismatch distribution analyses. Results from analysis of genetic diversity and neutrality statistics for Macronycteris commersoni clade B and C based on combined mtDNA sequences are presented in the table. Asterisks indicate results statistical significance (^∗^*P* < 0.05, ^∗∗^*P* < 0.01, ^∗∗∗^*P* < 0.001).

	Clade B	Clade C
Nucleotide diversity (*π*)	0.007	0.003
Haplotype diversity (Hd)	0.958	0.911
Fu and Li’s Fs	−63.055^∗∗∗^	−2.671
Tajima’s D	−1.811^∗^	−1.045
Ramos-Onsins and Rozas (R2)	0.0371	0.1253
Mismatch distribution	Unimodal	Bimodal

**Figure 6:**
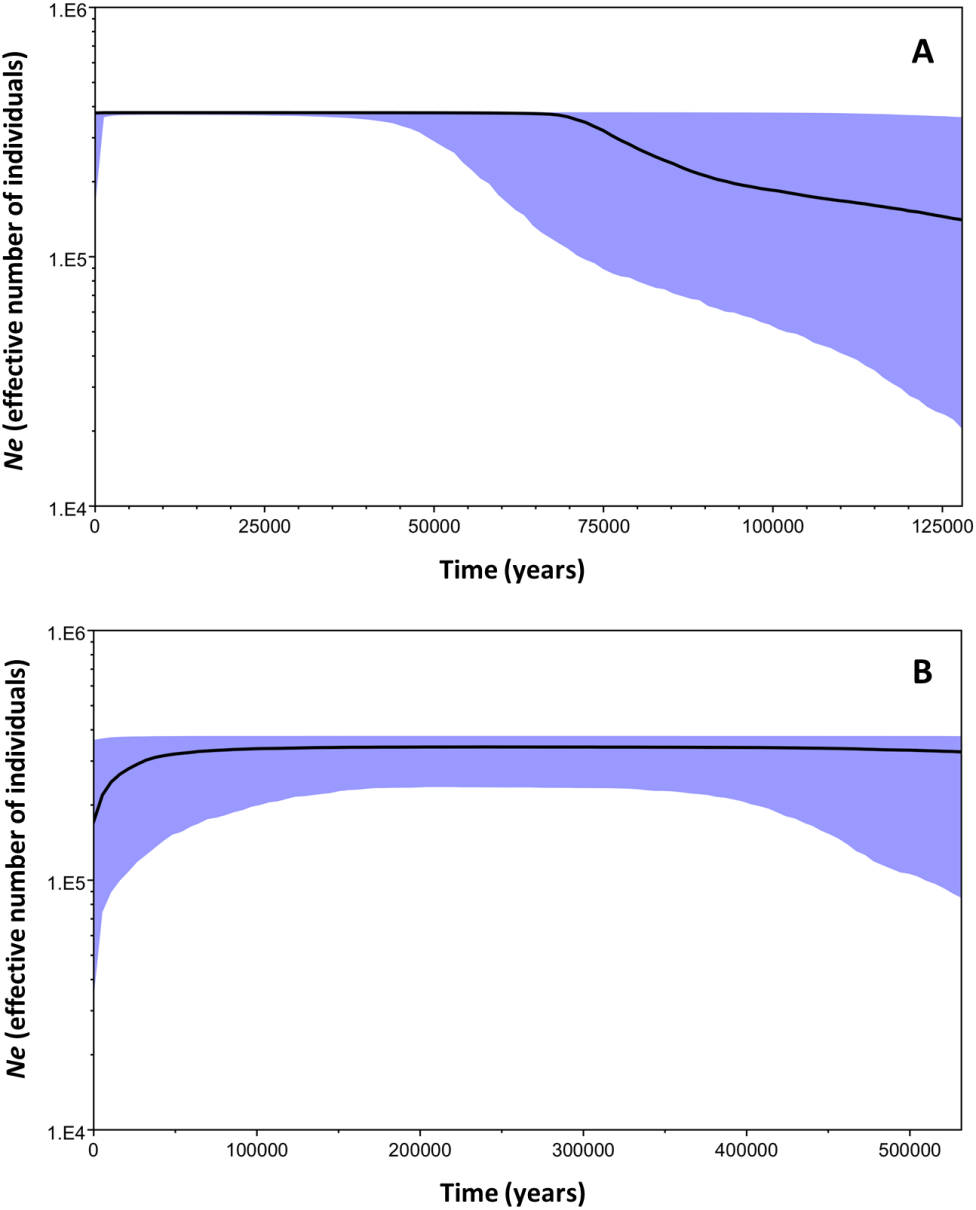
Bayesian skyline plots for the two mtDNA *Macronycteris commersoni* clades conducted using a strict molecular clock and based on a two year generation time. (A) clade B and (B) clade C. The thick solid line is the estimated median and the shaded area shows the 95% HPD limits.

The Bayesian Skyline plot analysis indicated that clade B underwent a slow demographic expansion that started ∼130,000 years ago, followed by stable growth starting at ∼70,000 years ago. The analysis showed no evidence of population decline during the evolutionary history of the clade. On the other hand, clade C has remained stable over time (Fig. 6).

## Discussion

The diversification of terrestrial faunal across Madagascar continues to receive attention (Herrera, 2017; Santini, Rojas & Donati, 2015). The processes responsible for this diversification are many. In addition to vicariance, climatic variability has been proposed as a major driver of lineage turnover on the island (Dewar & Richard, 2007). The climatic and biotic heterogeneity of Madagascar has led to the island being subdivided into discrete biogeographic zones that are characterized by distinct bioclimatic factors (Schatz, 2000), vegetation and elevation (Koechlin, 1972; Humbert, 1955) and faunal composition (Angel, 1942). The boundaries of these biogeographic zones also correspond to zoogeographic breaks common to different taxonomic groups (Koechlin, 1972; Angel, 1942; Martin, 1972). The population structure of *M. commersoni* provides an interesting scenario to test the link between bioclimatic variation and diversity as this species is widespread across the island and utilizes a wide range of habitat types including open woodland, degraded habitats, and forested areas from sea level to 1,325 m. Despite being widely distributed, there is evidence that *M. commersoni* exhibits morphological and bioacoustic variation across its geographical range, particularly along the western region of the island (Ramasindrazana et al., 2015). In this study we tested whether genetic variation within *M. commersoni* was shaped by the different bioclimatic zones.

### Phylogeography and demographic history

We recovered some geographic genetic subdivision within *M*. *commersoni*, with clade C restricted to the north of the island. Clades B and C diverged ∼0.82 MYA, in the mid-Pleistocene. We suggest that initial intraspecific divergence within *M*. *commersoni* might be related to refugial isolation, with taxa restricted to at least one zone that gave rise to clade C. The separation between lineages within clade B is less pronounced as members of this clade are distributed across the island. This pattern may have resulted from multi-directional dispersal during more favorable periods in the Pleistocene. Our results suggest that the expansion of the extant clade B commenced approximately 0.70 MYA. Few studies have estimated divergence dates of bat taxa below the family level, and most dates rely on a sparse fossil record (Teeling et al., 2005). Morphological convergence also complicates the use of fossil calibration in this group. Wesselman (1984) described fossil remains recovered from the Omo formation of Ethiopia and dated to the late Pliocene (2.08 MYA) as a distinct species, *H*. *kaumbului*. The author suggested that this taxon was morphologically similar to *H.* [*M*.] *commersoni*. However, recent molecular research has shown that many different taxa included in the *commersoni* group are morphologically very similar to each other (i.e., *M*. *gigas*, *M*. *vitattus*, *M*. *cryptovalorona* and *M*. *commersoni*), yet genetically quite distinct (Goodman et al., 2016; Rakotoarivelo et al., 2015). With this in mind, and given the uncertainty in estimation of the mutation rate of the *Cyt b*, the molecular dating results from this study should be treated with caution until verified calibration points can be added.

Nonetheless, this study indicates that *M*. *commersoni* exhibits genetic partitioning along a latitudinal gradient across Madagascar. At least three other Malagasy bat species, *Paratriaenops furculus* (Russell et al., 2007), *Chaerephon leucogaster* (Ratrimomanarivo et al., 2009a; Ratrimomanarivo et al., 2009b), and *Myotis goudoti* (Weyeneth, Goodman & Ruedi, 2011) show similar patterns of haplotypic segregation along this latitudinal gradient. The latitudinal distribution of different clades and the calculated expansion periods of these other species differ from late Pleistocene in *M. goudoti* to early Holocene in *C. leucogaster.* Whether this suggests lack of common historical processes underlying the different demographic events or if this disparity is the result of limitations of molecular dating remains to be clarified.

Intriguingly, the extinct *M*. *besaoka* described from the late Pleistocene-Holocene of Anjohibe Cave, western lowland Madagascar was temporally sympatric with *M*. *commersoni* (Samonds, 2007). No clear hypothesis has been presented on the principal factor that led to the extinction of *M*. *besaoka*. Changes in vegetational types in lowland areas of the western half of the island in the late Pleistocene and Holocene of Madagascar saw a shift to drier climates and more arid natural vegetational types (Burney, 1995; Burney, 1997; Goodman & Jungers, 2014). These changes were most notable in the extreme southwest during the late Holocene with shifts from forests and woodlands to drier wooded savanna (Burney, 1993; Goodman & Jungers, 2014). Similarly, northwestern Madagascar was the scene of vegetational changes from a mosaic of dry forest and wooded savanna from ∼3,500 years BP to savanna formation from 1,000–500 years BP (Matsumoto & Burney, 1994; Crowley & Samonds, 2013; Goodman & Jungers, 2014). What is not clear is why the factor(s) that led to the extinction of *M. besaoka* did not have the same impact on the presumed ecologically similar *M. commersoni*.

### Population genetic structure

The mtDNA haplotype network and phylogenetic analysis confirm the split between the northern clade C and clade B as reported by Rakotoarivelo et al. (2015). The present study extended thefinding of the latter study by providing addition evidence for the fine-scale phylogenetic structure of *M. commersoni* along the west coast of Madagascar.

The Bayesian clustering analyses revealed four genetically distinct groups. Clade C was also recovered as a separate genetic cluster in the population levels clustering analysis. Clade B was further subdivided into three genetic clusters (clusters 1, 3 and 4) by the BAPS analysis. The distribution of mtDNA haplotypes broadly follows the major bioclimatic breaks (Fig. 4). This fine-scale genetic structure is, however, shallow with 95% of the genetic variance occurring within populations. AMOVA did reveal a relatively low although highly significant proportion of the variance among groups of *M*. *commersoni* based on latitude (north vs south) and climate (degree of humidity). Significant genetic differences were also observed between populations from the dry region (Dry1 and Dry2) and subarid region (Table 4), suggesting that differences among the bioclimatic zones may be contributing towards the phylogeographic pattern observed.

African members of the *M*. *commersoni* species complex, specifically *M*. *vittatus* and *M. gigas*, undertake local seasonal migrations associated with fluctuations in prey abundance (McWilliam, 1982; Vaughan, 1977). Large hipposiderid bats have high wing loading and low to medium aspect ratios (Norberg & Rayner, 1987), which may favor relatively quick, long-distance movements, allowing individuals to track food resources (Jones & Rayner, 1989; Bernard & Fenton, 2003).

On Madagascar, seasonal presence of *M*. *commersoni* has been documented at Kirindy (CNFEREF) (Fig. 1) (Rakotondramanana & Goodman, 2011; Ramasindrazana et al., 2015), which is presumed to be associated with intra-island movements. This behavior, particularly at a broad geographical scale, may explain the shallow phylogeographic structure in this species. On the other hand, there is evidence from the northwest, specifically the region of Anjohibe, that local *M*. *commersoni* populations remain inactive in caves during times of resource shortage (AR Rakotoarivelo, 2010, unpublished data), which may explain the genetic uniqueness of clade C. Reher et al. (2018) reported similar torpor behavior in the Tsimanampetsotsa National Park in south-western Madagascar.

Genetically divergent populations have been recognized as conservation priorities (Palsböll, Berube & Allendorf, 2007; Lu et al., 2013). Based on this and the results recovered in the current study, the populations of *M*. *commersoni* belonging to clade C of Analamera, Ankarana, Montagne d’Ambre, and Marovaza (Fig. 1) are of conservation importance.

## Conclusions

Environmental variability has *shaped* the evolution and distribution of taxa on Madagascar (Muldoon & Goodman, 2010; Fuchs et al., 2016). Few studies, however, have focused on widely distributed species such as *M*. *commersoni*, for which barriers to gene flow are less obvious. This study provides evidence for several genetically distinct mtDNA lineages within *M*. *commersoni.* The distribution of the observed genetic variability follows the bioclimatic zones along the west coast of Madagascar. By focusing on a highly mobile species with broad distribution across the island, this study contributes towards a growing body of work highlighting that climatic variation has influenced the distribution of biodiversity on Madagascar.

##  Supplemental Information

10.7717/peerj.5866/supp-1 Table S1 List of specimens and associated Genbank accession numbers for the mtDNA control region (CR) and cytochrome b (Cyt b) sequences used in the present study PN, Parc National, RS, Réserve Spéciale, SF, Station Forestière. Collection numbers are the catalogue numbers of the respective museum: FMNH–Field Museum of Natural History, AMNH–American Museum of Natural History, and UADBA—Université d’Antananarivo, Département de Biologie Animale.

10.7717/peerj.5866/supp-2 Figure S1 Bayesian phylogram based on mtDNA control region (CR) and Cytochrome b (*Cyt b*) data drawn from 146 individual *Macronycteris commersoni* Nodal support values are represented as Bayesian posterior probability/likelihood bootstrap percent (∗,posterior probability values ≥0.50 and #, likelihood bootstrap percent ≥ 50).

## Additional Information and Declarations

### Data Availability

The following information was supplied regarding data availability: GenBank: KT371750–KT371770; KU302123–KU302248; KT896072–KT896197; KT583822–KT583830.

## References

[ref-1] Ali JR, Aitchison JC (2008). Gondwana to Asia: plate tectonics, paleogeography and the biological connectivity of the Indian sub-continent from the Middle Jurassic through latest Eocene (166–35 Ma). Earth-Science Reviews.

[ref-2] Ali JR, Huber M (2010). Mammalian biodiversity on Madagascar controlled by ocean currents. Nature.

[ref-3] Angel F (1942). Les lézards de Madagascar. Memoires de l’Académie Malgache.

[ref-4] Bernard E, Fenton MB (2003). Bat mobility and roosts in a fragmented landscape in Central Amazonia, Brazil. Biotropica.

[ref-5] Burney DA (1993). Late Holocene environmental change in arid southwestern Madagascar. Quaternary Research.

[ref-6] Burney DA, Lourenço WR (1995). Climate change and fire ecology as factors in the Quaternary biogeography of Madagascar. Biogéographie de Madagascar.

[ref-7] Burney DA, Goodman SM, Patterson BD (1997). Theories and facts regarding Holocene environmental change before and after human colonization. Natural change and human impact in Madagascar.

[ref-8] Chan LM, Goodman SM, Nowak M, Weisrock DW, Yoder AD (2011). Increased population sampling confirms low genetic divergence among *Pteropus* (Chiroptera: Pteropodidae) fruit bats of Madagascar and other western Indian Ocean islands [Internet]. PLOS Currents: Tree of Life.

[ref-9] Cheng L, Connor TR, Sirén J, Aanensen DM, Corander J (2013). Hierarchical and spatially explicit clustering of DNA sequences with BAPS software. Molecular Biology and Evololution.

[ref-10] Christidis L, Goodman SM, Naughton K, Appleton B (2014). Insights into the evolution of a cryptic radiation of bats: dispersal and ecological radiation of Malagasy *Miniopterus* (Chiroptera: Miniopteridae). PLOS ONE.

[ref-11] Cibois A, Slikas B, Schulenberg TS, Pasquet E (2001). An endemic radiation of Malagasy songbirds is revealed by mitochondrial DNA sequence data. Evolution.

[ref-12] Corander J, Marttinen P (2006). Bayesian identification of admixture events using multilocus molecular markers. Molecular Ecology.

[ref-13] Cornet A (1974). Essai de cartographie bioclimatique à Madagascar. Notice Explicative de l’ORSTOM.

[ref-14] Crowley BE, Samonds KE (2013). Stable carbon isotope values confirm a recent increase in grasslands in northwestern Madagascar. The Holocene.

[ref-15] Cruaud A, Raherilalao MJ, Pasquet E, Goodman SM (2011). Phylogeography and systematics of the Malagasy rock-thrushes (Muscicapidae, *Monticola*). Zoologica Scripta.

[ref-16] Darriba D, Taboada GL, Doallo R, Posada D (2012). jModelTest 2: more models, new heuristics and parallel computing. Nature Methods.

[ref-17] Dewar RE, Richard AF (2007). Evolution in the hypervariable environment of Madagascar. Proceedings of the National Academy of Sciences of the United States of America.

[ref-18] Drummond AJ, Rambaut A (2007). BEAST: Bayesian evolutionary analysis by sampling trees. BMC Evolutionary Biology.

[ref-19] Drummond AJ, Rambaut A, Shapiro B, Pybus OG (2005). Bayesian coalescent inference of past population dynamics from molecular sequences. Molecular Biology and Evolution.

[ref-20] Excoffier L, LischerH EL (2010). Arlequin suite ver 3.5: a new series of programs to perform population genetics analyses under Linux and Windows. Molecular Ecology Resources.

[ref-21] Felsenstein J (2005). PHYLIP: phylogeny inference package.

[ref-22] Foley NM, Thong VD, Soisook P, Goodman SM, Armstrong KN, Jacobs DS, Peuchmaille SJ, Teeling EC (2015). How and why overcome the impediments to resolution: lessons from rhinolophid and hipposiderid bats. Molecular Biology and Evolution.

[ref-23] Fu YX (1997). Statistical tests of neutrality of mutations against population growth, hitchhiking and background selection. Genetics.

[ref-24] Fuchs J, Lemoine D, Luis Parra J, Pons Marie J-M, Raherilalao J, Prys-Jones R, Thebaud C, Warren BH, Goodman SM (2016). Long-distance dispersal and inter-island colonization across the western Malagasy Region explain diversification in brush-warblers (Passeriformes: *Nesillas*). Biological Journal of the Linnean Society.

[ref-25] Fuchs J, Parra JL, Goodman SM, Raherilalao MJ, VanDerWal J, Bowie RCK (2013). Extending species distribution models to the past 120,000 years corroborates the lack of phylogeographic structure in the crested drongo (*Dicrurus forficatus*) from Madagascar. Biological Journal of the Linnean Society.

[ref-26] Fuchs J, Pons J-M, Pasquet E, Raherilalao MJ, Goodman SM (2007). Geographical structure of the genetic variation in the Malagasy scops-owl (*Otus rutilus* s.l.) inferred from mitochondrial sequence data. The Condor.

[ref-27] Goodman SM (2006). Hunting of Microchiroptera in extreme south-western Madagascar. Oryx.

[ref-28] Goodman SM, Chan LM, Nowak MD, Yoder AD (2010a). Phylogeny and biogeography of western Indian Ocean *Rousettus* (Chiroptera: Pteropodidae). Journal of Mammalogy.

[ref-29] Goodman SM, Jungers WL (2014). Extinct Madagascar: picturing the Island’s past.

[ref-30] Goodman SM, Maminirina CP, Bradman HM, Christidis L, Appleton B (2010b). Patterns of morphological and genetic variation in the endemic Malagasy bat *Miniopterus gleni* (Chiroptera: Miniopteridae), with the description of a new species, *M. griffithsi*. Journal of Zoological Systematics and Evolutionary Research.

[ref-31] Goodman SM, Ramasindrazana B, Goodman SM, Raherilalao MJ (2013). Bats or the order Chiroptera. Atlas of selected land vertebrates of Madagascar.

[ref-32] Goodman SM, Schoeman MC, Rakotoarivelo AR, Willows-Munro S (2016). How many species of *Hipposideros* have occurred on Madagascar since the Late Pleistocene?. Zoological Journal of the Linnean Society.

[ref-33] Hall TA (1999). BioEdit: a user-friendly biological sequence alignment editor and analysis program for Windows 95/98/NT. Nucleic Acids Symposium Series.

[ref-34] Harpending HC (1994). Signature of ancient population growth in a low resolution mitochondrial DNA mismatch distribution. Human Biology.

[ref-35] Harpending HC, Sherry ST, Rogers AR, Stoneking M (1993). The genetic structure of ancient human populations. Current Anthropology.

[ref-36] Herrera PJ (2017). Testing the adaptive radiation hypothesis for the lemurs of Madagascar. Royal Society Open Science.

[ref-37] Ho SYW, Shapiro B (2011). Skyline plot methods for estimating demographic history from nucleotide sequences. Molecular Ecology Resources.

[ref-38] Humbert H (1955). Les territoires phytogéographiques de Madagascar. Leur cartographie. Année Biologique.

[ref-39] Irwin DM, Kocher TD, Wilson AC (1991). Evolution of the cytochrome b gene of mammals. Journal of Molecular Evolution.

[ref-40] Jones G, Rayner MV, Hanák V, Horáček I, Gaisler J (1989). Optimal flight speed in pipistrelle bats, *Pipistrellus pipistrellus*. European bat research 1987.

[ref-41] Jønsson KA, Fabre P-H, Fritz SA, Etienne RS, Ricklefs RE, Jørgensen TB, Fjeldsa J, Rahbek C, Ericson PGP, Woog F, Pasquet E, Irestedt M (2012). Ecological and evolutionary determinants for the adaptive radiation of the Madagascan vangas. Proceedings of the National Academy of Sciences of the United States of America.

[ref-42] Koechlin J, Battistini R, Richard-Vendard G (1972). Flora and vegetation of Madagascar. Biogeography and ecology in Madagascar.

[ref-43] Lamb JM, Naidoo T, Taylor PJ, Napier M, Ratrimomanarivo F, Goodman SM (2012). Genetically and geographically isolated lineages of a tropical bat (Chiroptera, Molossidae) show demographic stability over the late Pleistocene. Biological Journal of the Linnean Society.

[ref-44] Lamb JM, Ralph TMC, Naidoo T, Taylor PJ, Ratrimomanarivo F, Stanley WT, Goodman SM (2011). Toward a molecular phylogeny for the Molossidae (Chiroptera) of Afro-Malagasy region. Acta Chiropterologica.

[ref-45] Librado P, Rozas J (2009). DnaSP ver. 5: a software for comprehensive analysis of DNA polymorphism data. Bioinformatics.

[ref-46] Liu T, Sun K, Park YC, Feng J (2016). Phylogenetic relationships and evolutionary history of the greater horseshoe bat, *Rhinolophus ferrumequinum*, in Northeast Asia. PeerJ.

[ref-47] Lu G, Lin A, Luo J, Blondel DV, Meiklejohn KA, Sun K, Feng J (2013). Phylogeography of the Ricketts big-footed bat, *Myotis pilosus* (Chiroptera: Vespertilionidae): a novel pattern of genetic structure of bats in China. BMC Evolutionary Biology.

[ref-48] Martin RD (1972). Adaptive radiation and behaviour of the Malagasy lemurs. Philosophical Transactions of the Royal Society of London (Series B).

[ref-49] Matsumoto K, Burney DA (1994). Late Holocene environments at Lake Mitsinjo, northwestern Madagascar. The Holocene.

[ref-50] McWilliam AN (1982). Adaptive responses to seasonality in four species of insectivorous bats in coastal Kenya. Ph.D. Thesis.

[ref-51] Miller MP (2005). Alleles in space (AIS): computer software for the joint analysis of interindividual spatial and genetic information. Journal of Heredity.

[ref-52] Muldoon K, Goodman SM (2010). Ecological biogeography of Malagasy non-volant mammals: community structure is correlated with habitat. Journal of Biogeography.

[ref-53] Myers N, Mittermeier R, Mittermeier C, Da Fonseca G, Kent J (2000). Biodiversity hotspots for conservation priorities. Nature.

[ref-54] Nabholz B, Glemin S, Galtier N (2008). Strong variations of mitochondrial mutation rate across mammals the longevity hypothesis. Molecular Biology and Evolution.

[ref-55] Noonan B, Chippindale PT (2006). Vicariant origin of Malagasy reptiles supports late Cretaceous Antarctic land bridge. American Naturalist.

[ref-56] Norberg UM, Rayner JMV (1987). Ecological morphology and flight in bats (Mammalia: Chiroptera): wing adaptations, flight performance, foraging strategy and echolocation. Philosophical Transactions of the Royal Society B: Biological Sciences.

[ref-57] Palsböll PJ, Berube M, Allendorf FW (2007). Identification of management units using population genetic data. Trends in Ecology and Evolution.

[ref-58] Pearson RG, Raxworthy CJ (2009). The evolution of local endemism in Madagascar: watershed versus climatic gradient hypotheses evaluated by null biogeographic models. Evolution.

[ref-59] Posada D, Crandall KA (1998). Modeltest: testing the model of DNA substitution. Bioinformatics.

[ref-60] Puechmaille SJ, Allegrini B, Boston ESM, Dubourg-Savage M-J, Evin A, Knochel A, Bris YL, Lecoq V, Lemaire M, Rist D, Teeling EC (2012). Genetic analyses reveal further cryptic lineages within the *Myotis nattereri* species complex. Mammalian Biology.

[ref-61] Raharinantenaina IMO, Kofoky AM, Mbohoahy T, Andriafidison D, Randrianandrianina F, Ramilijaona OR, Jenkins RKB (2008). *Hipposideros commersoni* (E. Geoffory, 1813, Hipposideridae) roosting in trees in littoral forest, south-eastern Madagascar. African Bat Conservation News.

[ref-62] Rakotoarivelo AR, Ralisata M, Ramilijaona OR, Rakotomalala MR, Racey PA, Jenkins RKB (2009). The food habits of a Malagasy giant: *Hipposideros commersoni* (E. Geoffroy, 1813). African Journal of Ecology.

[ref-63] Rakotoarivelo AR, Ranaivoson N, Ramilijaona OR, Kofoky AF, Racey PA, Jenkins RKB (2007). Seasonal food habits of five sympatric forest microchiropterans in western Madagascar. Journal of Mammalogy.

[ref-64] Rakotoarivelo AR, Willows-Munro S, Schoeman MC, Lamb JM, Goodman SM (2015). Cryptic diversity in *Hipposideros commersoni* sensu stricto (Chiroptera: Hipposideridae) in the western portion of Madagascar. BMC Evolutionary Biology.

[ref-65] Rakotondramanana CF, Goodman SM (2011). Inventaire de chauves-souris dans la concession forestiere de Kirindy CNFEREF, Morondava, Madagascar. Malagasy Nature.

[ref-66] Ramasindrazana B, Rakotondramanana CF, Schoeman MC, Goodman SM (2015). Evidence of echolocation call divergence in *Hipposideros commersoni* sensu stricto (E. Geoffroy, 1803) from Madagascar and correlation with body size. Acta Chiropterologica.

[ref-67] Rambaut A, Suchard MA, Xie D, Drummond AJ (2014). http://beast.bio.ed.ac.uk/Tracer.

[ref-68] Ramos-Onsins SE, Rozas J (2002). Statistical properties of new neutrality tests against population growth. Molecular Biology and Evolution.

[ref-69] Ranivo J, Goodman SM (2007). Variation géographique de *Hipposideros commersoni* de la zone sèche de Madagascar (Mammalia, Chiroptera, Hipposideridae). Verhandlungen des Naturwissenschaftlichen Vereins in Hamburg.

[ref-70] Ratrimomanarivo F, Goodman SM, Hoosen N, Taylor PJ, Lamb J (2008). Morphological and molecular variation in *Mops leucostigma* (Chiroptera: Molossidae) of Madagascar and the Comoros: phylogeny, phylogeography and geographic variation. Mitteilungen aus dem Zoologischen Museum Hamburg.

[ref-71] Ratrimomanarivo FH, Goodman SM, Stanley WT, Naidoo T, Taylor PJ, Lamb J (2009b). Geographic and phylogeographic variation in *Chaerephon leucogaster* (Chiroptera: Molossidae) of Madagascar and the western Indian Ocean islands of Mayotte and Pemba. Acta Chiropterologica.

[ref-72] Ratrimomanarivo FH, Goodman SM, Taylor PJ, Melson B, Lamb J (2009a). Morphological and genetic variation in *Mormopterus jugularis* (Chiroptera: Molossidae) in different bioclimatic regions of Madagascar with natural history notes. Mammalia.

[ref-73] Ratrimomanarivo F, Vivian J, Goodman SM, Lamb J (2007). Morphological and molecular assessment of the specific status of *Mops midas* (Chiroptera: Molossidae) from Madagascar and Africa. African Zoology.

[ref-74] Razakarivony V, Rajemison B, Goodman SM (2005). The diet of Malagasy Microchiroptera based on stomach contents. Mammalian Biology.

[ref-75] Reddy S, Driskell A, Rabosky DL, Hackett SJ, Schulenberg TS (2012). Diversification and the adaptive radiation of the vangas of Madagascar. Proceedings of the Royal Society B: Biological Sciences.

[ref-76] Reher S, Ehlers J, Rabarison H, Dausmann KH (2018). Short and hyperthermic torpor responses in the Malagasy bat Macronycteris commersoni reveal a broader hypometabolic scope in heterotherms. Journal of Comparative Physiology B.

[ref-77] Ronquist F, Teslenko M, Van der Mark P, Ayres D, Darling A, Höhna S, Liu L, Suchard MA, Huelsenbeck JP (2012). MrBayes 3.2: efficient Bayesian phylogenetic inference and model choice across a large model space. Systematic Biology.

[ref-78] Russell AL, Goodman SM, Cox MP (2008). Coalescent analyses support multiple mainland-to-island dispersals in the evolution of Malagasy *Triaenops* bats (Chiroptera: Hipposideridae). Journal of Biogeography.

[ref-79] Russell AL, Goodman SM, Fiorentino I, Yoder AD (2008). Population genetic analysis of *Myzopoda* (Chiroptera: Myzopodidae) in Madagascar. Journal of Mammalogy.

[ref-80] Russell AL, Ranivo J, Palkovacs EP, Goodman SM, Yoder AD (2007). Working at the interface of phylogenetics and population genetics: a biogeographical analysis of *Triaenops* spp. (Chiroptera: Hipposideridae). Molecular Ecology.

[ref-81] Salzburger W, Ewing GB, Von Haeseler A (2011). The performance of phylogenetic algorithms in estimating haplotype genealogies with migration. Molecular Ecology.

[ref-82] Samonds KE (2007). Late Pleistocene bat fossils from Anjohibe Cave, northwestern Madagascar. Acta Chiropterologica.

[ref-83] Samonds KE, Godfrey LR, Ali JR, Goodman SM, Vences M, Sutherland MR, Irwin MT, Krause DW (2012). Spatial and temporal arrival patterns of Madagascar’s vertebrate fauna explained by distance, ocean currents, and ancestor type. Proceedings of the National Academy of Sciences of the United States of America.

[ref-84] Samonds KE, Godfrey LR, Ali JR, Goodman SM, Vences M, Sutherland MR, Irwin MT, Krause DW (2013). Imperfect isolation: factors and filters shaping Madagascar’s extant vertebrate fauna. PLOS ONE.

[ref-85] Santini L, Rojas D, Donati G (2015). Evolving through day and night: origin and diversification of activity pattern in modern primates. Behavioral Ecology.

[ref-86] Schatz GE, Lourenço WR, Goodman SM (2000). Endemism in the Malagasy tree flora. Diversité et Endémisme à Madagascar.

[ref-87] Schoeman MC, Goodman SM, Ramasindrazana B, Koubinova D (2015). Species interactions during diversification and community assembly in Malagasy *Miniopterus* bats. Evolutionary Ecology.

[ref-88] Tajima F (1989). Statistical method for testing the neutral mutation hypothesis by DNA polymorphism. Genetics.

[ref-89] Tamura K, Stecher G, Peterson D, Filipski A, Kumar S (2013). MEGA6: molecular evolutionary genetics analysis version 6.0. Molecular Biology and Evolution.

[ref-90] Teeling EC, Springer MS, Madsen O, Bates P, O’Brien SJ, Murphy WJ (2005). A molecular phylogeny for bats illuminates biogeography and the fossil record. Science.

[ref-91] Thompson JD, Gibson TJ, Plewniak F, Jeanmougin F, Higgins DG (1997). The CLUSTAL X windows interface: flexible strategies for multiple sequence alignment aided by quality analysis tools. Nucleic Acids Research.

[ref-92] Thong VD, Puechmaille SJ, Denzinger A, Bates PJJ, Dietz C, Csorba G, Soisook P, Teeling EC, Matsumura S, Furey NM, Schnitzler HU (2012). Systematics of the *Hipposideros turpis* complex and a description of a new subspecies from Vietnam. Mammal Review.

[ref-93] Vaughan TA (1977). Foraging behaviour of the giant leaf-nosed bat (*Hipposideros commersoni*). East African Wildlife Journal.

[ref-94] Vences M, Wollenberg KC, Vieites DR, Lees DC (2009). Madagascar as a model region of species diversification. Trends in Ecology & Evolution.

[ref-95] Wesselman HB, Hecht MK, Szalay FS (1984). The Omo micromammals: systematics and paleoecology of early man sites from Ethiopia. Contributions to vertebrate evolution.

[ref-96] Weyeneth N, Goodman SM, Ruedi M (2011). Do diversification models of Madagascar’s biota explain the population structure of the endemic bat *Myotis goudoti* (Chiroptera: Vespertilionidae)?. Journal of Biogeography.

[ref-97] Wilkinson GS, Chapman AM (1991). Length and sequence variation in evening bat d-loop mtDNA. Genetics.

[ref-98] Wilmé L, Goodman SM, Ganzhorn JU (2006). Biogeographic evolution of Madagascar’s microendemic biota. Science.

[ref-99] Yoder AD, Nowak MD (2006). Has vicariance or dispersal been the predominant force in Madagascar? Only time will tell. Annual Review of Ecology and Systematics.

[ref-100] Zwickl DJ (2006). Genetic algorithm approaches for the phylogenetic analysis of large biological sequence datasets under the maximum likelihood criterion. Ph.D. dissertation.

