# Supplementary material for: Phylogeography and population genetics of the endemic Malagasy bat, Macronycteris commersoni s.s. (Chiroptera: Hipposideridae)
Source: PeerJ. 2019 Jan 17;7:e5866. doi: 10.7717/peerj.5866 (PMC6339777; doi:10.7717/peerj.5866)
Supplement: Table S1 — PN, Parc National, RS, Réserve Spéciale, SF, Station Forestière. Collection numbers are the catalogue numbers of the respective museum: FMNH–Field Museum of Natural History, AMNH–American Museum of Natural History, and UADBA—Université d’Antananarivo, Département de Biologie Animale. [file peerj-07-5866-s001.docx]

**S1 Table:** List of specimens and associated Genbank accession numbers for the mtDNA control region (CR) and cytochrome b (*Cyt b*) sequences used in the present study. PN = Parc National, RS = Réserve Spéciale, SF = Station Forestière. Collection numbers are the catalogue numbers of the respective museum: FMNH - Field Museum of Natural History, AMNH -American Museum of Natural History, and UADBA - Université d’Antananarivo, Département de Biologie Animale.

| **Species** | **Specimen number** | **Province/Locality** | **Latitude** | **Longitude** | **Year of collection** | **Sex** | **GenBank numbers** | |
| --- | --- | --- | --- | --- | --- | --- | --- | --- |
|  |  |  |  |  |  |  | **CR** | ***Cyt b*** |
| *M. commersoni* | FMNH 183978 | Antsiranana/Lac Sahaka, Foret d’Analabe | -13.0571 | 49.904 | 2004 | Female | KU302200 | KT896149 |
| *M. commersoni* | FMNH 183979 | Antsiranana/Lac Sahaka, Foret d’Analabe | -13.1136 | 49.9121 | 2004 | Male | KU302201 | KT896150 |
| *M. commersoni* | FMNH 183980 | Antsiranana/Montagne de Français, Foret d’Ampitiliantsambo | -12.3371 | 49.3845 | 2005 | Female | KT371766 | KT5838013 |
| *M. commersoni* | FMNH 183981 | Antsiranana/Montagne de Français, Foret d’Ampitiliantsambo | -12.3371 | 49.3845 | 2005 | Female | KU302208 | KT896157 |
| *M. commersoni* | FMNH 188574 | Antsiranana/Nosy Be, Centre National de Recherches Océanographiques, 1.5 km E Hell-ville | -13.4067 | 48.2917 | 2006 | Male | KU302190 | KT896139 |
| *M. commersoni* | UADBA 32987 | Antsiranana/PN d’Ankarana, 2.6 km E Andrafiabe, in forest near Andrafiabe Cave | -13.9317 | 49.0567 | 2012 | Female | KT371768 | KT5838014 |
| *M. commersoni* | UADBA 32988 | Antsiranana/PN d’Ankarana, 2.6 km E Andrafiabe, in forest near Andrafiabe Cave | -13.9317 | 49.0567 | 2012 | Female | KU302234 | KT896183 |
| *M. commersoni* | UADBA 32989 | Antsiranana/PN d’Ankarana, 2.6 km E Andrafiabe, in forest near Andrafiabe Cave | -13.9317 | 49.0567 | 2012 | Female | KU302235 | KT896184 |
| *M. commersoni* | FMNH 221307 | Antsiranana/PN d’Ankarana, 2.6 km E Andrafiabe, in forest near Andrafiabe Cave | -13.9317 | 49.0567 | 2012 | Female | KU302236 | KT896185 |
| *M. commersoni* | FMNH 221308 | Antsiranana/PN d’Ankarana, 2.6 km E Andrafiabe, in forest near Andrafiabe Cave | -13.9317 | 49.0567 | 2012 | Female | KT371769 | KT5838029 |
| *M. commersoni* | FMNH 169722 | Antsiranana/PN d’Ankarana, 2.6 km E Andrafiabe, near Andrafiabe Cave | -12.9317 | 49.0567 | 2001 | Female | KU302124 | KT896073 |
| *M. commersoni* | FMNH 169707 | Antsiranana/PN d’Ankarana, 2.6 km E Andrafiabe, near Andrafiabe Cave | -12.9317 | 49.0567 | 2001 | Female | KT371750 | KT5838022 |
| *M. commersoni* | FMNH 169708 | Antsiranana/PN d’Ankarana, 2.6 km E Andrafiabe, near Andrafiabe Cave | -12.9317 | 49.0567 | 2001 | Female | KU302130 | KT896079 |
| *M. commersoni* | FMNH 213588 | Antsiranana/PN d’Ankarana, 2.6 km E Andrafiabe, near Andrafiabe Cave | -12.9317 | 49.0567 | 2010 | Female | KU302196 | KT896145 |
| *M. commersoni* | FMNH 177385 | Antsiranana/PN d’Ankarana, 3.5 km ESE Andrafiabe, Grotte d’Andrafiabe | -12.9357 | 49.0573 | 2003 | Male | KU302197 | KT896146 |
| *M. commersoni* | FMNH 177386 | Antsiranana/PN d’Ankarana, 3.5 km ESE Andrafiabe, Grotte d’Andrafiabe | -12.9357 | 49.0573 | 2003 | Male | KU302198 | KT896147 |
| *M. commersoni* | FMNH 177387 | Antsiranana/PN d’Ankarana, 3.5 km ESE Andrafiabe, Grotte d’Andrafiabe | -12.9357 | 49.0573 | 2003 | Female | KU302199 | KT896148 |
| *M. commersoni* | FMNH 176277 | Antsiranana/PN d’Ankarana, 3.5 km SE Andrafiabe (village) | -12.9417 | 49.055 | 2003 | Male | KU302163 | KT896112 |
| *M. commersoni* | FMNH 156340 | Antsiranana/PN de la Montagne d’Ambre, Grande Lac, 12 km SW Joffreville | -12.5967 | 49.16 | 1996 | Female | KU302123 | KT896072 |
| *M. commersoni* | FMNH 154601 | Antsiranana/PN de la Montagne d’Ambre, 5.5 km SW Joffreville | -12.5272 | 49.1717 | 1995 | Female | KU302129 | KT896078 |
| *M. commersoni* | FMNH 178803 | Antsiranana/RS d’Analamerana, Grotte de Bazaribe, 3.6 km SE Menagisy | -12.7121 | 49.4735 | 2004 | Female | KU302165 | KT896114 |
| *M. commersoni* | FMNH 178804 | Antsiranana/RS d’Analamerana, Grotte de Bazaribe, 3.6 km SE Menagisy | -12.7121 | 49.4735 | 2004 | Female | KU302166 | KT896115 |
| *M. commersoni* | FMNH 178805 | Antsiranana/RS d’Analamerana, Grotte de Bazaribe, 3.6 km SE Menagisy | -12.7121 | 49.4735 | 2004 | Female | KU302167 | KT896116 |
| *M. commersoni* | FMNH 178806 | Antsiranana/RS d’Analamerana, Grotte de Bazaribe, 3.6 km SE Menagisy | -12.7121 | 49.4735 | 2004 | Female | KT371755 | KT5838016 |
| *M. commersoni* | FMNH 178807 | Antsiranana/RS d’Analamerana, Grotte de Bazaribe, 3.6 km SE Menagisy | -12.7121 | 49.4735 | 2004 | Female | KU302168 | KT896117 |
| *M. commersoni* | FMNH 178808 | Antsiranana/RS d’Analamerana, Grotte de Bazaribe, 3.6 km SE Menagisy | -12.7121 | 49.4735 | 2004 | Female | KT371756 | KT5838017 |
| *M. commersoni* | FMNH 178809 | Antsiranana/RS d’Analamerana, Grotte de Bazaribe, 3.6 km SE Menagisy | -12.7121 | 49.4735 | 2004 | Female | KT371757 | KT5838018 |
| *M. commersoni* | FMNH 178810 | Antsiranana/RS d’Analamerana, Grotte de Bazaribe, 3.6 km SE Menagisy | -12.7121 | 49.4735 | 2004 | Female | KT371758 | KT5838019 |
| *M. commersoni* | FMNH 178811 | Antsiranana/RS d’Analamerana, Grotte de Bazaribe, 3.6 km SE Menagisy | -12.7121 | 49.4735 | 2004 | Female | KT371759 | KT5838020 |
| *M. commersoni* | FMNH 178812 | Antsiranana/RS d’Analamerana, Grotte de Bazaribe, 3.6 km SE Menagisy | -12.7121 | 49.4735 | 2004 | Female | KU302169 | KT896118 |
| *M. commersoni* | FMNH 178813 | Antsiranana/RS d’Analamerana, Grotte de Bazaribe, 3.6 km SE Menagisy | -12.7121 | 49.4735 | 2004 | Female | KU302170 | KT896119 |
| *M. commersoni* | FMNH 178814 | Antsiranana/RS d’Analamerana, Grotte de Bazaribe, 3.6 km SE Menagisy | -12.7121 | 49.4735 | 2004 | Female | KU302171 | KT896120 |
| *M. commersoni* | FMNH 178815 | Antsiranana/RS d’Analamerana, Grotte de Bazaribe, 3.6 km SE Menagisy | -12.7121 | 49.4735 | 2004 | Female | KT371760 | KT5838021 |
| *M. commersoni* | FMNH 151706 | Fianarantsoa/Ambalavao, 43 km S, Andringitra Reserve | -22.2278 | 47.0036 | 1993 | Female | KU302128 | KT896077 |
| *M. commersoni* | FMNH 175962 | Fianarantsoa/just outside PN de l’Isalo, along Menamaty River, 8 km N Ranohira (RN7) | -22.4856 | 45.3917 | 2002 | Female | KU302144 | KT896093 |
| *M. commersoni* | FMNH 175963 | Fianarantsoa/just outside PN de l’Isalo, along Menamaty River, 8 km N Ranohira (RN7) | -22.4856 | 45.3917 | 2002 | Female | KU302145 | KT896094 |
| *M. commersoni* | FMNH 175964 | Fianarantsoa/just outside PN de l’Isalo, along Menamaty River, 8 km N Ranohira (RN7) | -22.4856 | 45.3917 | 2002 | Female | KU302146 | KT896095 |
| *M. commersoni* | FMNH 175965 | Fianarantsoa/just outside PN de l’Isalo, along Menamaty River, 8 km N Ranohira (RN7) | -22.4856 | 45.3917 | 2002 | Female | KU302147 | KT896096 |
| *M. commersoni* | FMNH 175966 | Fianarantsoa/just outside PN de l’Isalo, along Menamaty River, 8 km N Ranohira (RN7) | -22.4856 | 45.3917 | 2002 | Female | KT371751 | KT5838023 |
| *M. commersoni* | FMNH 175967 | Fianarantsoa/just outside PN de l’Isalo, along Menamaty River, 8 km N Ranohira (RN7) | -22.4856 | 45.3917 | 2002 | Female | KU302148 | KT896097 |
| *M. commersoni* | FMNH 217940 | Fianarantsoa/PN de l’Isalo, 10,5 km SW Ranohira, Hotel Jardin du Roi | -22.31 | 45.29 | 2011 | Female | KT371767 | KT5838031 |
| *M. commersoni* | UADBA 50267 | Fianarantsoa/PN de l’Isalo, 3,8 km NW Ranohira along Namaza River | -22.31 | 45.29 | 2011 | Male | KU302221 | KT896170 |
| *M. commersoni* | FMNH 218012 | Fianarantsoa/PN de l’Isalo, 3,8 km NW Ranohira along Namaza River | -22.31 | 45.29 | 2011 | Female | KU302222 | KT896171 |
| *M. commersoni* | UADBA 50268 | Fianarantsoa/PN de l’Isalo, 3,8 km NW Ranohira along Namaza River | -22.3167 | 45.2933 | 2002 | Female | KU302223 | KT896172 |
| *M. commersoni* | FMNH 175968 | Fianarantsoa/PN de l’Isalo, along Sahanafa River, 28 km SE Berenty-Betsileo | -22.3167 | 45.2933 | 2002 | Male | KU302149 | KT896098 |
| *M. commersoni* | FMNH 175969 | Fianarantsoa/PN de l’Isalo, along Sahanafa River, 28 km SE Berenty-Betsileo | -22.3167 | 45.2933 | 2002 | Female | KU302150 | KT896099 |
| *M. commersoni* | FMNH 175971 | Fianarantsoa/PN de l’Isalo, along Sahanafa River, 28 km SE Berenty-Betsileo | -22.3167 | 45.2933 | 2002 | Female | KU302151 | KT896100 |
| *M. commersoni* | FMNH 175972 | Fianarantsoa/PN de l’Isalo, along Sahanafa River, 28 km SE Berenty-Betsileo | -22.3167 | 45.2933 | 2002 | Female | KU302152 | KT896101 |
| *M. commersoni* | FMNH 175973 | Fianarantsoa/PN de l’Isalo, along Sahanafa River, 28 km SE Berenty-Betsileo | -22.3167 | 45.2933 | 2002 | Female | KU302153 | KT896102 |
| *M. commersoni* | FMNH 175974 | Fianarantsoa/PN de l’Isalo, along Sahanafa River, 28 km SE Berenty-Betsileo | -22.3167 | 45.2933 | 2002 | Female | KU302154 | KT896103 |
| *M. commersoni* | FMNH 175975 | Fianarantsoa/PN de l’Isalo, along Sahanafa River, 28 km SE Berenty-Betsileo | -22.3167 | 45.2933 | 2002 | Male | KU302155 | KT896104 |
| *M. commersoni* | FMNH 175976 | Fianarantsoa/PN de l’Isalo, along Sahanafa River, 28 km SE Berenty-Betsileo | -22.3167 | 45.2933 | 2002 | Male | KU302156 | KT896105 |
| *M. commersoni* | FMNH 175977 | Fianarantsoa/PN de l’Isalo, along Sahanafa River, 28 km SE Berenty-Betsileo | -22.3167 | 45.2933 | 2002 | Male | KU302157 | KT896106 |
| *M. commersoni* | FMNH 175961 | Fianarantsoa/PN de l’Isalo, Canyon des Singes (Andranokova), 2 km W Ranohira bas | -22.4867 | 45.3783 | 2002 | Female | KU302143 | KT896092 |
| *M. commersoni* | UADBA 50270 | Fianarantsoa/PN de l’Isalo, Grotte de Bekapity | -23.6333 | 45.2181 | 2012 | Female | KU302224 | KT896173 |
| *M. commersoni* | UADBA 50271 | Fianarantsoa/PN de l’Isalo, Grotte de Bekapity | -23.6333 | 45.2181 | 2012 | Female | KU302225 | KT896174 |
| *M. commersoni* | UADBA 50272 | Fianarantsoa/PN de l’Isalo, Grotte de Bekapity | -23.6333 | 45.2181 | 2012 | Female | KU302226 | KT896175 |
| *M. commersoni* | UADBA 50273 | Fianarantsoa/PN de l’Isalo, Grotte de Bekapity | -23.6333 | 45.2181 | 2012 | Female | KU302227 | KT896176 |
| *M. commersoni* | UADBA 50274 | Fianarantsoa/PN de l’Isalo, Grotte de Bekapity | -23.6333 | 45.2181 | 2012 | Female | KU302228 | KT896177 |
| *M. commersoni* | FMNH 218016 | Fianarantsoa/PN de l’Isalo, Grotte de Bekapity | -23.6333 | 45.2181 | 2012 | Female | KU302229 | KT896178 |
| *M. commersoni* | FMNH 218017 | Fianarantsoa/PN de l’Isalo, Grotte de Bekapity | -23.6333 | 45.2181 | 2012 | Female | KU302230 | KT896179 |
| *M. commersoni* | FMNH 218018 | Fianarantsoa/PN de l’Isalo, Grotte de Bekapity | -23.6333 | 45.2181 | 2012 | Female | KU302231 | KT896180 |
| *M. commersoni* | FMNH 218019 | Fianarantsoa/PN de l’Isalo, Grotte de Bekapity | -23.6333 | 45.2181 | 2012 | Female | KU302232 | KT896181 |
| *M. commersoni* | FMNH 218020 | Fianarantsoa/PN de l’Isalo, Grotte de Bekapity | -23.6333 | 45.2181 | 2012 | Female | KU302233 | KT896182 |
| *M. commersoni* | FMNH 184029 | Mahajanga/4.2 km SE Marovaza, in cave | -14.966 | 47.308 | 2005 | Female | KU302189 | KT896138 |
| *M. commersoni* | FMNH 184030 | Mahajanga/Berivotra, village | -14.966 | 47.308 | 2005 | Female | KT371765 | KT5838012 |
| *M. commersoni* | FMNH 184886 | Mahajanga/Berivotra, village | -15.904 | 46.5979 | 2005 | Female | KU302204 | KT896153 |
| *M. commersoni* | FMNH 184887 | Mahajanga/Berivotra, village | -15.904 | 46.5979 | 2005 | Female | KU302205 | KT896154 |
| *M. commersoni* | FMNH 187786 | Mahajanga/Forêt d’Ampidirabe, 16.3 km N Antsalova | -18.531 | 44.5939 | 2006 | Male | KU302214 | KT896163 |
| *M. commersoni* | FMNH 209236 | Mahajanga/Forêt de Beanka, cave along Kimanambolo River | -18.063 | 44.5411 | 2009 | Male | KU302195 | KT896144 |
| *M. commersoni* | FMNH 187784 | Mahajanga/Forêt de Mamakibetro, 18.3 km NNE Ansalova | -18.516 | 44.6603 | 2006 | Female | KU302212 | KT896161 |
| *M. commersoni* | FMNH 187785 | Mahajanga/Forêt de Mamakibetro, 18.3 km NNE Ansalova | -18.516 | 44.6603 | 2006 | Female | KU302213 | KT896162 |
| *M. commersoni* | FMNH 209108 | Mahajanga/Grotte d’Anjohibe, 3.7 km NE Antanamarina | -15.538 | 46.886 | 2008 | Female | KU302191 | KT896140 |
| *M. commersoni* | FMNH 209109 | Mahajanga/Grotte d’Anjohibe, 3.7 km NE Antanamarina | -15.538 | 46.886 | 2008 | Female | KU302192 | KT896141 |
| *M. commersoni* | FMNH 209110 | Mahajanga/Grotte d’Anjohibe, 3.7 km NE Antanamarina | -15.538 | 46.886 | 2008 | Male | KU302193 | KT896142 |
| *M. commersoni* | FMNH 209111 | Mahajanga/Grotte d’Anjohibe, 3.7 km NE Antanamarina | -15.538 | 46.886 | 2008 | Male | KU302194 | KT896143 |
| *M. commersoni* | UADBA 32916 | Mahajanga/Grotte d’Anjohibe, 3.7 km NE Antanamarina | -15.538 | 46.886 | 2012 | Female | KT371770 | KT5838030 |
| *M. commersoni* | FMNH 184027 | Mahajanga/Grotte d’Ankelimahogo, 18.5 km S Anjajavy village | -15.19 | 47.2029 | 2004 | Male | KU302179 | KT896128 |
| *M. commersoni* | FMNH 184028 | Mahajanga/Marovaza | -14.947 | 47.2746 | 2005 | Female | KU302188 | KT896137 |
| *M. commersoni* | FMNH 175776 | Mahajanga/PN du Tsingy de Namoroka, near source of Mandevy River, 32 km NW Andranomavo | -16.38 | 45.345 | 2002 | Female | KU302138 | KT896087 |
| *M. commersoni* | FMNH 175777 | Mahajanga/PN du Tsingy de Namoroka, near source of Mandevy River, 32 km NW Andranomavo | -16.38 | 45.345 | 2002 | Female | KT371750 | KT5838022 |
| *M. commersoni* | FMNH 175778 | Mahajanga/PN du Tsingy de Namoroka, near source of Mandevy River, 32 km NW Andranomavo | -16.38 | 45.345 | 2002 | Female | KU302139 | KT896088 |
| *M. commersoni* | FMNH 178558 | Mahajanga/PN du Tsingy de Namoroka, Site Andriabe, 2 km SE Namoroka village | -16.407 | 45.3067 | 2003 | Female | KU302164 | KT896113 |
| *M. commersoni* | FMNH 194580 | Mahajanga/RS d’Ambohijanahary, Mahajeby Forest, 21.8 km ESE de Beravina | -18.27 | 45.4056 | 2006 | Female | KU302215 | KT896164 |
| *M. commersoni* | FMNH 177299 | Mahajanga/SF d’Ampirojoa | -16.315 | 46.81 | 2003 | Male | KU302160 | KT896109 |
| *M. commersoni* | FMNH 177300 | Mahajanga/SF d’Ampirojoa | -16.315 | 46.81 | 2003 | Male | KU302161 | KT896110 |
| *M. commersoni* | FMNH 177301 | Mahajanga/SF d’Ampirojoa | -16.315 | 46.81 | 2003 | Male | KU302162 | KT896111 |
| *M. commersoni* | FMNH 177302 | Mahajanga/SF d’Ampirojoa | -16.315 | 46.81 | 2003 | Male | KT371754 | KT5838025 |
| *M. commersoni* | FMNH 183982 | Toamasina/Foulepointe, Forêt d’Analalava | -17.702 | 49.4586 | 2005 | Male | KU302202 | KT896151 |
| *M. commersoni* | FMNH 183983 | Toamasina/Marovaza, in cave | -17.702 | 49.4586 | 2005 | Male | KU302203 | KT896152 |
| *M. commersoni* | FMNH 179200 | Toamasina/SF de Tampolo, 10 km NW Fenerive-Est | -17.28 | 49.42 | 2004 | Female | KU302172 | KT896121 |
| *M. commersoni* | FMNH 179201 | Toamasina/SF de Tampolo, 10 km NW Fenerive-Est | -17.28 | 49.42 | 2004 | Male | KU302173 | KT896122 |
| *M. commersoni* | FMNH 179202 | Toamasina/SF de Tampolo, 10 km NW Fenerive-Est | -17.28 | 49.42 | 2004 | Male | KU302174 | KT896123 |
| *M. commersoni* | FMNH 194633 | Toliara/District de Manja, Beronto Forest, 11 km SSE Ankiliabo | -21.779 | 43.9778 | 2007 | Male | KU302216 | KT896165 |
| *M. commersoni* | FMNH 194634 | Toliara/District de Manja, Beronto Forest, 11 km SSE Ankiliabo | -21.779 | 43.9778 | 2007 | Male | KU302217 | KT896166 |
| *M. commersoni* | FMNH 176489 | Toliara/Fiherenana | -23.238 | 43.8731 | 2002 | Male | KU302206 | KT896155 |
| *M. commersoni* | FMNH 176156 | Toliara/Forêt des Mikea, 16 km W Vorehy | -22.267 | 43.4783 | 2003 | Female | KU302209 | KT896158 |
| *M. commersoni* | FMNH 176157 | Toliara/Forêt des Mikea, 16 km W Vorehy | -22.267 | 43.4783 | 2003 | Female | KU302210 | KT896159 |
| *M. commersoni* | FMNH 176164 | Toliara/Forêt des Mikea, 7.5 km NE Tsifota | -22.8 | 43.4333 | 2003 | Female | KU302158 | KT896107 |
| *M. commersoni* | FMNH 176163 | Toliara/Forêt des Mikea, 7.5 km NE Tsifota | -22.8 | 43.4333 | 2003 | Female | KU302159 | KT896108 |
| *M. commersoni* | FMNH 176158 | Toliara/Forêt des Mikea, 8.4 km SSE Befandefa | -22.217 | 43.33 | 2003 | Female | KU302211 | KT896160 |
| *M. commersoni* | FMNH 176155 | Toliara/Forêt des Mikea, 9.5 km W Ankiloaka | -22.778 | 43.5233 | 2003 | Male | KT371753 | KT5838024 |
| *M. commersoni* | FMNH 184169 | Toliara/Grotte d’Androimpano, 4.2 km NE Itampolo (village), on old road to Ejeda | -24.65 | 43.9633 | 2005 | Female | KU302180 | KT896129 |
| *M. commersoni* | FMNH 184170 | Toliara/Grotte d’Androimpano, 4.2 km NE Itampolo (village), on old road to Ejeda | -24.65 | 43.9633 | 2005 | Female | KT371763 | KT5838028 |
| *M. commersoni* | FMNH 184171 | Toliara/Grotte d’Androimpano, 4.2 km NE Itampolo (village), on old road to Ejeda | -24.65 | 43.9633 | 2005 | Female | KU302181 | KT896130 |
| *M. commersoni* | FMNH 184172 | Toliara/Grotte d’Androimpano, 4.2 km NE Itampolo (village), on old road to Ejeda | -24.65 | 43.9633 | 2005 | Female | KU302182 | KT896131 |
| *M. commersoni* | FMNH 184174 | Toliara/Grotte d’Androimpano, 4.2 km NE Itampolo (village), on old road to Ejeda | -24.65 | 43.9633 | 2005 | Female | KU302183 | KT896132 |
| *M. commersoni* | FMNH 184175 | Toliara/Grotte d’Androimpano, 4.2 km NE Itampolo (village), on old road to Ejeda | -24.65 | 43.9633 | 2005 | Female | KU302184 | KT896133 |
| *M. commersoni* | FMNH 184176 | Toliara/Grotte d’Androimpano, 4.2 km NE Itampolo (village), on old road to Ejeda | -24.65 | 43.9633 | 2005 | Female | KU302185 | KT896134 |
| *M. commersoni* | FMNH 184177 | Toliara/Grotte d’Androimpano, 4.2 km NE Itampolo (village), on old road to Ejeda | -24.65 | 43.9633 | 2005 | Male | KU302186 | KT896135 |
| *M. commersoni* | FMNH 184178 | Toliara/Grotte d’Androimpano, 4.2 km NE Itampolo (village), on old road to Ejeda | -24.65 | 43.9633 | 2005 | Male | KU302187 | KT896136 |
| *M. commersoni* | UADBA 32378 | Toliara/Grotte de Tanambao, 0.75 km E St. Augustin | -23.549 | 43.7674 | 2012 | Female | KU302219 | KT896168 |
| *M. commersoni* | UADBA 32379 | Toliara/Grotte de Tanambao, 0.75 km E St. Augustin | -23.549 | 43.7674 | 2012 | Male | KU302220 | KT896169 |
| *M. commersoni* | UADBA 33692 | Toliara/Kirindy Forest (CNFEREF) | -20.079 | 44.6778 | 2012 | Male | KU302237 | KT896186 |
| *M. commersoni* | UADBA 33693 | Toliara/Kirindy Forest (CNFEREF) | -20.079 | 44.6778 | 2012 | Male | KU302238 | KT896187 |
| *M. commersoni* | UADBA 33694 | Toliara/Kirindy Forest (CNFEREF) | -20.079 | 44.6778 | 2012 | Male | KU302239 | KT896188 |
| *M. commersoni* | UADBA 33695 | Toliara/Kirindy Forest (CNFEREF) | -20.079 | 44.6778 | 2012 | Male | KU302240 | KT896189 |
| *M. commersoni* | UADBA 33696 | Toliara/Kirindy Forest (CNFEREF) | -20.079 | 44.6778 | 2012 | Male | KU302241 | KT896190 |
| *M. commersoni* | UADBA 33697 | Toliara/Kirindy Forest (CNFEREF) | -20.079 | 44.6778 | 2012 | Male | KU302242 | KT896191 |
| *M. commersoni* | UADBA 33698 | Toliara/Kirindy Forest (CNFEREF) | -20.079 | 44.6778 | 2012 | Male | KU302243 | KT896192 |
| *M. commersoni* | UADBA 33699 | Toliara/Kirindy Forest (CNFEREF) | -20.076 | 44.6747 | 2012 | Male | KU302244 | KT896193 |
| *M. commersoni* | UADBA 33700 | Toliara/Kirindy Forest (CNFEREF) | -20.079 | 44.6778 | 2012 | Male | KU302245 | KT896194 |
| *M. commersoni* | UADBA 33701 | Toliara/Kirindy Forest (CNFEREF) | -20.079 | 44.6778 | 2012 | Male | KU302246 | KT896195 |
| *M. commersoni* | UADBA 33702 | Toliara/Kirindy Forest (CNFEREF) | -20.079 | 44.6778 | 2012 | Female | KU302247 | KT896196 |
| *M. commersoni* | FMNH 222747 | Toliara/Kirindy Forest (CNFEREF) | -20.076 | 44.6747 | 2012 | Female | KU302248 | KT896197 |
| *M. commersoni* | UADBA 32376 | Toliara/Mikea, Grotte de Maki near Hôtel La Mangrove | -23.472 | 43.7707 | 2012 | Female | KU302218 | KT896167 |
| *M. commersoni* | FMNH 176052 | Toliara/PN de Kirindy Mite, 0.75 km SW Manahy | -20.868 | 43.9075 | 2002 | Female | KU302140 | KT896089 |
| *M. commersoni* | FMNH 176053 | Toliara/PN de Kirindy Mite, 0.75 km SW Manahy | -20.868 | 43.9075 | 2002 | Female | KU302141 | KT896090 |
| *M. commersoni* | FMNH 176054 | Toliara/PN de Kirindy Mite, 0.75 km SW Manahy | -20.868 | 43.9075 | 2002 | Female | KU302142 | KT896091 |
| *M. commersoni* | FMNH 173151 | Toliara/PN de Tsimanampetsotsa, 6.5 km NE Efoetse, near Mitoho Cave | -24.05 | 43.75 | 2002 | Female | KU302136 | KT896085 |
| *M. commersoni* | FMNH 173162 | Toliara/PN de Tsimanampetsotsa, 6.5 km NE Efoetse, near Mitoho Cave | -24.05 | 43.75 | 2002 | Female | KU302137 | KT896086 |
| *M. commersoni* | FMNH 183929 | Toliara/PN de Tsimanampetsotsa, 6.5 km NE Efoetse, near Mitoho Cave | -24.05 | 43.75 | 2004 | Female | KU302175 | KT896124 |
| *M. commersoni* | FMNH 183930 | Toliara/PN de Tsimanampetsotsa, 6.5 km NE Efoetse, near Mitoho Cave | -24.05 | 43.75 | 2004 | Female | KU302176 | KT896125 |
| *M. commersoni* | FMNH 183931 | Toliara/PN de Tsimanampetsotsa, 6.5 km NE Efoetse, near Mitoho Cave | -24.05 | 43.75 | 2004 | Female | KU302177 | KT896126 |
| *M. commersoni* | FMNH 183932 | Toliara/PN de Tsimanampetsotsa, 6.5 km NE Efoetse, near Mitoho Cave | -24.05 | 43.75 | 2004 | Female | KT371761 | KT583826 |
| *M. commersoni* | FMNH 183933 | Toliara/PN de Tsimanampetsotsa, 6.5 km NE Efoetse, near Mitoho Cave | -24.05 | 43.75 | 2004 | Female | KU302178 | KT896127 |
| *M. commersoni* | FMNH 183934 | Toliara/PN de Tsimanampetsotsa, 6.5 km NE Efoetse, near Mitoho Cave | -24.05 | 43.75 | 2004 | Female | KT371762 | KT5838027 |
| *M. commersoni* | FMNH 172760 | Toliara/PN du Tsingy de Bemaraha, 3.5 km E Bekopaka | -19.14 | 44.8283 | 2001 | Male | KU302135 | KT896084 |
| *M. commersoni* | FMNH 172745 | Toliara/PN du Tsingy de Bemaraha, Ankidrodroa, 2.5 km NE Bekopaka | -19.132 | 44.8083 | 2001 | Male | KU302125 | KT896074 |
| *M. commersoni* | FMNH 172747 | Toliara/PN du Tsingy de Bemaraha, Ankidrodroa, 2.5 km NE Bekopaka | -19.132 | 44.8083 | 2001 | Female | KU302126 | KT896075 |
| *M. commersoni* | FMNH 172748 | Toliara/PN du Tsingy de Bemaraha, Ankidrodroa, 2.5 km NE Bekopaka | -19.132 | 44.8083 | 2001 | Female | KU302127 | KT896076 |
| *M. commersoni* | FMNH 172755 | Toliara/PN du Tsingy de Bemaraha, Ankidrodroa, 2.5 km NE Bekopaka | -19.132 | 44.8083 | 2001 | Male | KU302131 | KT896080 |
| *M. commersoni* | FMNH 172756 | Toliara/PN du Tsingy de Bemaraha, Ankidrodroa, 2.5 km NE Bekopaka | -19.132 | 44.8083 | 2001 | Male | KU302132 | KT896081 |
| *M. commersoni* | FMNH 172757 | Toliara/PN du Tsingy de Bemaraha, Ankidrodroa, 2.5 km NE Bekopaka | -19.132 | 44.8083 | 2001 | Male | KU302133 | KT896082 |
| *M. commersoni* | FMNH 172758 | Toliara/PN du Tsingy de Bemaraha, Ankidrodroa, 2.5 km NE Bekopaka | -19.132 | 44.8083 | 2001 | Male | KU302134 | KT896083 |
| *M. commersoni* | FMNH 177371 | Toliara/Ranobe | -23.04 | 43.6103 | 2003 | Male | KU302207 | KT896156 |
