# Supplementary figures and images for: Phylogeography and population genetics of the endemic Malagasy bat, Macronycteris commersoni s.s. (Chiroptera: Hipposideridae)
Source: PeerJ. 2019 Jan 17;7:e5866. doi: 10.7717/peerj.5866 (PMC6339777; doi:10.7717/peerj.5866)

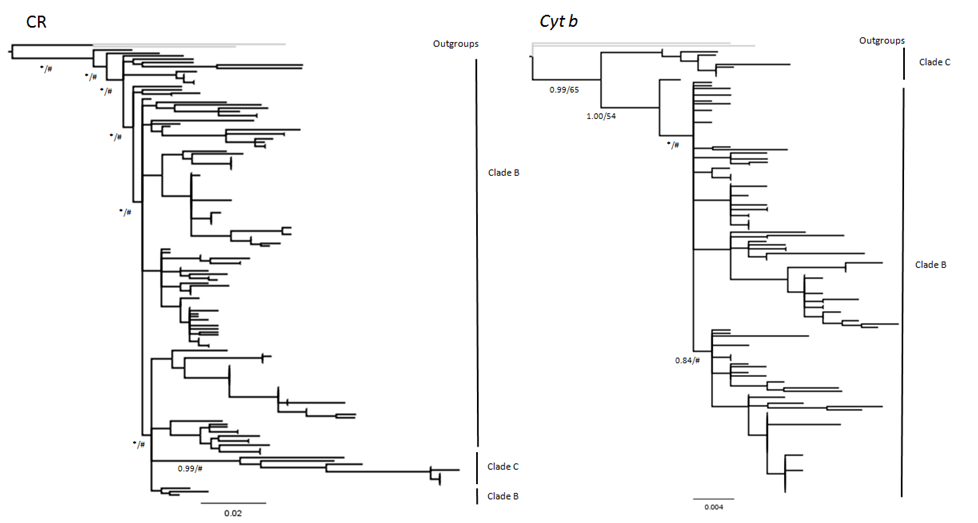

Supplement: Figure S1 — Nodal support values are represented as Bayesian posterior probability/likelihood bootstrap percent (∗,posterior probability values ≥0.50 and #, likelihood bootstrap percent ≥ 50). [file peerj-07-5866-s002.png]
